# Phosphoinositides regulate the TCR/CD3 complex membrane dynamics and activation

**DOI:** 10.1038/s41598-018-23109-8

**Published:** 2018-03-21

**Authors:** Nassima Chouaki Benmansour, Kilian Ruminski, Anne-Marie Sartre, Marie-Claire Phelipot, Audrey Salles, Elise Bergot, Ambroise Wu, Gaëtan Chicanne, Mathieu Fallet, Sophie Brustlein, Cyrille Billaudeau, Anthony Formisano, Sébastien Mailfert, Bernard Payrastre, Didier Marguet, Sophie Brasselet, Yannick Hamon, Hai-Tao He

**Affiliations:** 1 Aix Marseille Univ, CNRS, INSERM, CIML, Centre d’Immunologie de Marseille-Luminy, Marseille, France; 2 Institut des Maladies Métaboliques et Cardiovasculaires, Inserm U1048, Université Toulouse 3, Toulouse, France; 3 Laboratoire d’Hématologie, Centre Hospitalier Universitaire de Toulouse, Toulouse, France; 4 Aix-Marseille Univ, CNRS, Centrale Marseille, Institut Fresnel, UMR 7249, 13397 Marseille, France; 5 Present Address: UTechS Photonic BioImaging (Imagopole) Citech, Institut Pasteur, Paris, 75724 France; 6 Present Address: Micalis Institute, INRA, AgroParisTech, Université Paris-Saclay, 78350 Jouy-en-Josas, France

**Keywords:** Membrane lipids, Membrane structure and assembly, Cell signalling, Signal transduction

## Abstract

Phosphoinositides (PIs) play important roles in numerous membrane-based cellular activities. However, their involvement in the mechanism of T cell receptor (TCR) signal transduction across the plasma membrane (PM) is poorly defined. Here, we investigate their role, and in particular that of phosphatidylinositol 4,5-bisphosphate [PI(4,5)P2] in TCR PM dynamics and activity in a mouse T-cell hybridoma upon ectopic expression of a PM-localized inositol polyphosphate-5-phosphatase (Inp54p). We observed that dephosphorylation of PI(4,5)P2 by the phosphatase increased the TCR/CD3 complex PM lateral mobility prior stimulation. The constitutive and antigen-elicited CD3 phosphorylation as well as the antigen-stimulated early signaling pathways were all found to be significantly augmented in cells expressing the phosphatase. Using state-of-the-art biophotonic approaches, we further showed that PI(4,5)P2 dephosphorylation strongly promoted the CD3ε cytoplasmic domain unbinding from the PM inner leaflet in living cells, thus resulting in an increased CD3 availability for interactions with Lck kinase. This could significantly account for the observed effects of PI(4,5)P2 dephosphorylation on the CD3 phosphorylation. Our data thus suggest that PIs play a key role in the regulation of the TCR/CD3 complex dynamics and activation at the PM.

## Introduction

T cell activation is a central event of the adaptive immunity, which is initiated upon the T cell recognition of antigen by the T cell receptor (TCR)^1,2^. The TCR of the αβ T cells is a multichain polypeptide complex composed of a ligand recognition module with the heterodimer of TCRα and β subunits and a signal transducing module with invariant CD3ε, γ, δ, ζ subunits^3^. Upon TCR binding to its peptide-major histocompatibility complex (pMHC) ligand, one of the earliest and essential signaling is phosphorylation of the immunoreceptor tyrosine-based activation motifs (ITAMs), located in the cytoplasmic domain of the CD3 subunits, by Lck kinase. This phosphorylation results in the recruitment and activation of ZAP-70 kinase, which in turn couples the TCR phosphorylation to downstream signaling cascades by phosphorylating critical signaling intermediates^3,4^. However, one major hole has remained in this picture despite extensive studies carried out over the last three decades, which is the lack of understanding on the mechanism by which the ligand engagement of TCR/CD3 at the cell surface brings about the phosphorylation of CD3 ITAMs by Lck located at the inner leaflet of the plasma membrane (PM), a process that is commonly called TCR triggering^1^. Since Lck is known to be constitutively activated in the PM of resting T cells^5^, we could also ask why are the CD3 ITAMs only phosphorylated upon TCR engagement by ligand, and not in the resting state.

Different models have been proposed to explain the TCR triggering mechanism, and it is in fact possible that they represent different facets of the same mechanism, happening either simultaneously or sequentially^6–9^. Among these models, the conformational change model is one that has gained substantial interest in recent years^7,9^. It proposes that pMHC binding to TCR induces, through an unknown mechanism, the conformation/orientation change of the cytoplasmic domain of CD3 subunits (specially CD3ε and CD3ζ), converting it from a “close” to an “open” configuration in order to be phosphorylated by Lck. In the CD3ε cytoplasmic domain (CD3εCD), two other motifs are located at the N-terminus of the ITAM, which are the lipid-interacting basic-rich stretch (BRS)^9^ and Nck-interacting proline-rich sequence (PRS)^7^. In the CD3ζCD, one BRS is also present upstream of each of the 3 ITAMs. Beside to understand their functional roles, the study of interactions of the PRS and BRS with their respective ligands have been conducted to examine the conformation/orientation change of CD3εCD during TCR triggering^7,9^.

At the mechanistical level, the conformation change model suggests that for CD3ε, which is a central player for the activation of the TCR/CD3 complex, interactions between the positively charged BRS and negatively charged acidic phospholipids results in tight binding of CD3εCD to the PM inner leaflet causing the ITAM to be buried in the lipid bilayer^9–11^. Upon TCR engagement with pMHC, CD3εCD is released from the PM inner leaflet due BRS unbinding with lipids, allowing the ITAM to become available as a substrate of Lck^9,12^. However, this model has been challenged since the mutation of CD3ε BRS to prevent its binding to anionic lipids did not lead to the “expected” increase of CD3 phosphorylation either prior or upon TCR stimulation^13^. Nevertheless, new studies now show that the lack observed phosphorylation increase is presumably due to the fact that the mutation also causes a concomitant disruption of the constitutive interactions between CD3ε BRS and Lck^14,15^. On the other hand, the factors triggering the BRS unbinding from the PM inner leaflet upon TCR engagement is still a matter of speculation, but two elements are often being considered: the dynamic modifications of the local lipid environment, and the force of a torque exerted on the CD3ε chain by TCR-pMHC binding^16^.

PI(4,5)P2 and its main precursor, phosphatidylinositol 4-phosphate (PI4P) are the most abundant PIs of the PM. PI(4,5)P2 constitutes about 1% of the total PM phospholipids^17^ and is kown to play a pivotal role in numerous membrane-based cellular functions^18,19^ including membrane trafficking, membrane-cytoskeleton adhesion, as well as structural organization and function of transmembrane proteins such as ion channels, transporters and receptors^18,19^. PI(4,5)P2 is also involved in cell signaling cascade, acting as an important second messenger. Receptor-stimulated activation of phospholipase C (PLC) results in the hydrolysis of PI(4,5)P2 to generate inositol 1,4,5-trisphosphate (IP3) and diacylglycerol (DAG)^20^, which in turn activates the intracellular Ca^2+^ mobilization, and PKC/Ras signaling pathway, respectively. PI(4,5)P2 also is the source for the synthesis of phosphatidylinositol 3,4,5-trisphosphate PI(3,4,5)P3 by phosphatidylinositol 3-kinase, a key player in cell proliferation, differentiation, survival, migration and metabolic responses^21^. In fact, the signaling function of PI(4,5)P2 has largely been documented in T cell activation. Recent studies also have documented the role of PI(4,5)P2 in the T cell cytoskeleton organization and dynamics^1,2^. PI(4,5)P2 has reportedly been found not homogenously distributed across the membranes, but segregated into distinct membrane pools^22,23^ or membrane nano-assemblies^24^. This is consistent with the wide range of cellular functions of PI(4,5)P2^18,19^.

Here, we investigate the role of PI(4,5)P2 in the regulation of the TCR dynamics and activation at the PM by expression of a PM-targeted Inp54p that selectively dephosphorylates PI(4,5)P2 at this cellular compartment. In addition, based on the previous findings of CD3ε BRS binding to various PIs *in vitro*, we have also assessed the contribution of PI(4,5)P2 in the BRS-mediated association of CD3εCD with the PM inner leaflet in living T cells. Our data show that PI(4,5)P2 dephosphorylation led to an increased PM lateral mobility in the TCR, and this was parallel to an increase of the constitutive and ligand-independent phosphorylation of CD3. In cells expressing the phosphatase, TCR activation and early signaling pathway initiation upon engagement with peptide-major histocompatibility complex (pMHC), but not with anti-CD3 mAb, were found significantly augmented. Finally, using a combination of state-of-the-art biophotonic approaches including excitation-polarization-resolved fluorescence microscopy and spot-variaton fluorescence correlation spectroscopy, we provide evidence that PI(4,5)P2 dephosphorylation strongly altered the CD3ε cytoplasmic domain binding to the PM inner surface in live cells, and this event could significantly account in the observed modification on the TCR lateral dynamics and activation at the PM.

## Results

### The hydrolysis of PM PI(4,5)P2 in 3A9m T cells upon expression of PM-targeted Inp54p

**Figure 1:**
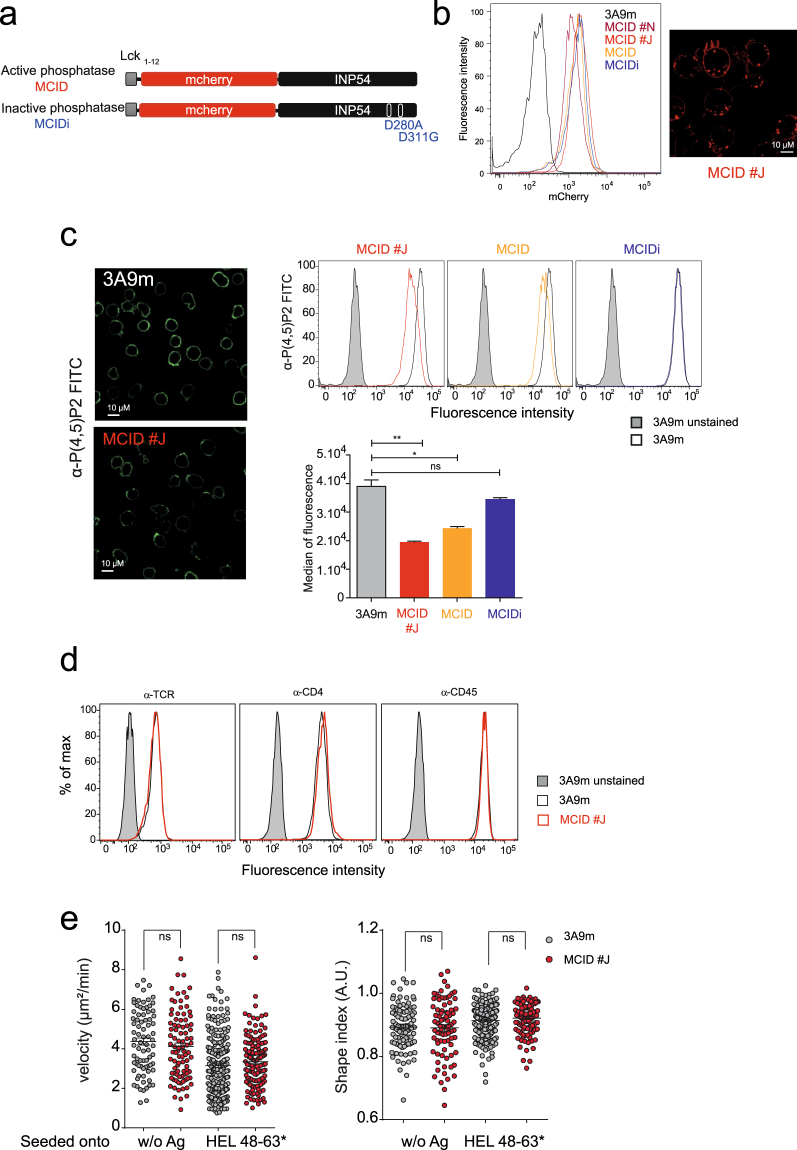
The hydrolysis of PM PI(4,5)P2 in 3A9m T cells upon expression of PM-targeted Inp54p. (**a**) The yeast phosphatase Inp54p is fused to mCherry downstream of the first 12 amino acids of Lck (MCID). As a control, an inactive phosphatase called inactive phosphatase (MCIDi) was generated, where the active aspartic acid sites at positions 280 and 311 were mutated to alanine and glutamic acid respectively (D280A, D311G). The ER retention site was deleted in order to avoid ER phosphatase localization. (**b**) Cells stablely expressing MCID were sorted by flow cytometry based on their cell surface receptor and mCherry expression. Clones displaying a monomodal level of mCherry fluorescence (left panel) expressed at the plasma membrane and in endosomal compartments were assessed by confocal microscopy as illustrated for MCID clone J (MCID #J) (right panel), Scale bar, 10 µm. (**c**) 3A9m mock-transfected WT or MCID-positive cells were immunostained with a FITC-conjugated anti-PI(4,5)P2 antibody. Confocal microscopy shows the PI(4,5)P2 labeling in 3A9m or MCID #J cells (scale bar, 10 µm) (left panel). Cells also were analyzed by flow cytometry (upper right panel). Overlays of the FACS profile were as followed: gray histograms for auto-fluorescence, black line for PI(4,5)P2 staining of 3A9m cells, orange line for 3A9m MCID cells, red line for MCID #J cells, and blue line for MCIDi cells. The medians of fluorescence were quantified for each cell type and plotted on a histogram (lower right panel) (n = 5). (**d**) TCR Cβ, CD4 and CD45 surface expression of 3A9m or MCID #J cells (in red) were quantified by flow cytometry with FITC-coupled specific mAbs. Unstained controls were shown in gray. The results are representative of five independent experiments. (**e**) The velocity and the shape index were quantified as indicated in Methods for 3A9m or MCID #J cells loaded with BD PBX calcium indicator and seeded onto COS-A^k^ and COS-A^k^_48–63_* APC cells, respectively.

To evaluate the role of PI(4,5)P2 in TCR signaling, we expressed a chimeric molecule in 3A9m T-cell hybridoma cells consisting of a yeast 5-PI phosphatase Inp54p^25^ that specifically dephosphorylates PI(4,5)P2 to produce PI4P, deleted of its ER retention C-terminal domain and an mCherry fluorescent protein linked to a short PM localization peptide Lck_1–12_ at the N-terminus (referred to as MCID) (Fig. 1a). Lck_1–12_ corresponds to the first twelve amino acid residues of Lck, which provides an efficient and robust signal for permanent targeting to the PM inner leaflet, owing to its one myristoylation and two palmitoylation groups^26^. The TCR of 3A9m cells specifically recognizes hen egg lysozyme (HEL) peptides bound to the MHCII molecule I-A^k ^^27^. Upon transfection, a stable MCID-positive cell population (referred as 3A9m MCID cells) was obtained based on their high expression of the chimera (Fig. 1b). Different individual clones expressing the MCID were subsequently established upon cell cloning of the bulk cell population. Among them, clones #J and #N (referred as MCID #J and #N) that exhibited normal surface level of TCR signaling molecules, including the TCR/CD3 complex, CD4 co-receptor and CD45 tyrosine phosphatase (Fig. 1d) were then used together with 3A9m MCID cells in our study.

Using a previously described immunostaining method^28,29^ that allows the specific and exclusive labeling of PI(4,5)P2 and PI4P at the PM, we found that the expression of MCID caused a strong reduction of PM PI(4,5)P2 level in 3A9m cells (Fig. 1c). This was a consequence of the PI(4,5)P2 hydrolysis because the expression of the chimera with an enzymatically inactive form of Inp54p^30^ (MCIDi) (Fig. 1a) showed no effects on the levels of PM PI(4,5)P2 (Fig. 1c, right panel). However, we did not observe a significant change in the PM PI4P in 3A9m cells in the presence or absence of MCID expression (Figure S1A,B). Further studies will be required to determine whether such a finding reflected a true constant level of PM PI4P upon MCID expression or if an increase in the amount of PM PI4P in MCID-expressing cells was not detected because it was present in a labile fraction largely lost during the sample preparation. We also conducted an analysis of total cellular steady-state labeling of ^32^P-PIs (Figure S1C) in 3A9m cells, which showed that the radioactivity incorporated in PI(4,5)P2 (^32^P-PI(4,5)P2) at steady-state normally was 5 to 6 times higher compared to that incorporated in PI4P (^32^P-PI4P). The MCID expression leaded to a strong decrease of ^32^P-PI(4,5)P2 (∼70%) and a 4-fold increase of ^32^P-PI4P, confirming that the enzyme actively hydrolyzed PI(4,5)P2 in 3A9m T cells.

Finally, using various techniques, we showed that the F-actin cortex was not grossly modified upon MCID expression (Figure S2A–C), although the possible local changes in the actin cytoskeleton organization could not be excluded. This observation was further corroborated by the unchanged cell shape index and averaged motility with or without antigen stimulation (Fig. 1e). If these findings were somehow unexpected, as PI(4,5)P2 has been implicated in the cortical actin skeleton organization^22,31^, the same kinds of observations also have previously been made in other studies with leukocyte cell types^32,33^. Hence, we propose that either the remaining amounts of PI(4,5)P2 in the MCID-expressing 3A9m cells was of sufficient to support its cortical cytoskeleton organizing function, or the MCID only hydrolyzed a specific pool of PI(4,5)P2 in the PM that was not directly involved in the overall cellular F-actin content^22^.

### The MCID expression results in the augmentation of CD3 phosphorylation and pMHC-stimulated TCR signaling initiation

**Figure 2:**
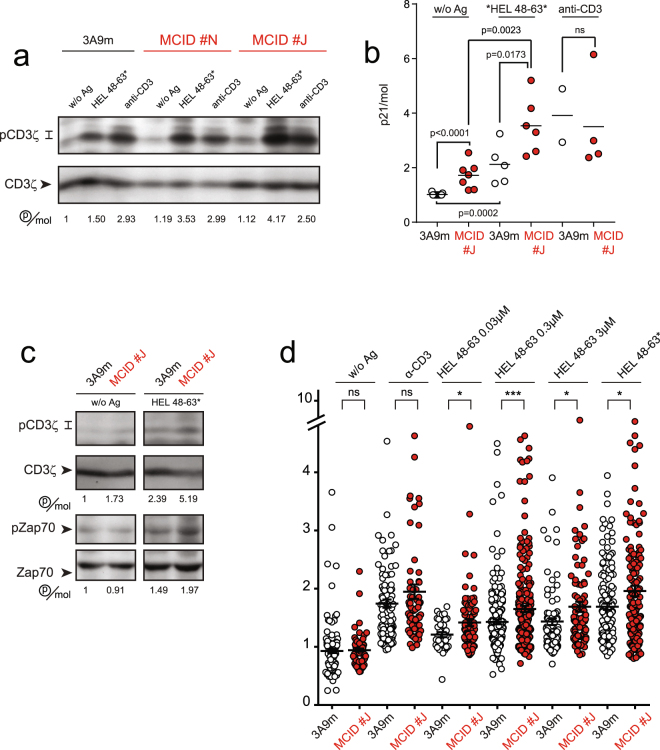
The MCID expression results in the increased TCR activation and signaling. (**a**) 3A9m mock-transfected WT cells or MCID #N or #J clones were seeded onto COS-A^k^ or COS-A^k^_48–63_* cells, or incubated with soluble anti-CD3ε mAb (145–2C11, 10 µg/ml), for 5 min at 37 °C. Cell lysates were immunoblotted with an anti-p-Tyr antibody (4G10) to detect the phosphorylated isoforms of CD3ζ (p21 and p23) or with an anti-CD3ζ antibody to detect the (p16) CD3ζ molecule, and the p21/CD3ζ ratios were quantified with a Bioimage analyzer LAS-1000. Cropped blots were shown for clarity and the uncropped blot images are displayed in Supplementary Figure S3A. (**b**) Statistical analysis of the p21/CD3ζ ratio from the immunoblot studies were performed as in (**a**). (**c**) Same as in (**a**), except that an additional analysis on the phosphorylation of Zap-70 (pTyr 319) also was included. Cropped blots were shown for clarity and the uncropped immunoblot images are displayed in Supplementary Figure S3B. (**d**) The Ca^2+^ store release analysis. T cells were loaded with PBX dye before being seeded onto APCs in presence of the CRAC blocker 2-APB (10 µM) and the fluorescence amplitude of individual cells over the threshold. T cells were stimulated with either the surface-immobilized anti-CD3ε mAb, or COS-A^k^ cells pulsed with various concentrations of HEL48–63 peptide or COS-A^k^_48–63_*.

To determine the role of PI(4,5)P2 in the regulation of activation of the TCR/CD3 and proximal signaling, we first examined the tyrosine phosphorylation of the CD3, whose stimulation constitutes one of the first signaling events upon TCR-ligand engagement. We used COS-7 cells transfected with the α and β chains of the MHC class II I-A^k^ (designated as COS-A^k^ cells) as antigen presenting cells (APCs). The TCR of 3A9m T cells was stimulated with either COS-A^k^ cells pulsed with HEL_48–63_ peptide, or with COS-7 cells by expressing I-A^k^ covalently linked to HEL48–63 (designated as COS-A^k^_48–63_*). The analysis of CD3ζ phosphorylation indicated a notable and statistically significant increased activation TCR upon binding to pMHC ligand HEL_48–63_/I-A^k^ in both MCID #J and #N clones (Fig. 2a,b), as compared to wild-type (WT) 3A9m cells. The same increase also was observed in 3A9m MCID, but not MCIDi cells (Figure S4A,B). In contrast, similar increase was not seen when the TCR was stimulated with soluble anti-CD3 mAb (Figs 2a,b, S4A,B), despite unchanged binding efficiency (Figure S6A). This finding indicated that the effects on TCR activation observed with MCID was not due to a general augmentation of the signaling pathway activation. Our analysis further revealed that the increase in basal CD3ζ phosphorylation in the 3A9m MCID and MCID #J cells also was statistically significant compared to WT cells (Figs 2a,b, S4A,B). We next examined the activation of ZAP-70 kinase by examining the phosphorylation status of its tyrosine 319, which is one of the most critical steps in the TCR signaling initiation^34^. Data from this analysis clearly showed an increased phosphorylation of ZAP-70 in MCID-expressing cells (Fig. 2c), confirming that dephosphorylation of PI(4,5)P2 resulted in enhanced TCR activation and signaling initiation.

### The MCID expression leads to the enhanced TCR-triggered Ca^2+^ store release upon pMHC stimulation

To further confirm the role of PI(4,5)P2 in the regulation of the TCR triggering, we examined the increase in [Ca^2+^]_i_ in living 3A9m T cells, as this was immediately down-stream to the proximal tyrosine kinase cascades upon TCR stimulation^35^. As a consequence of the protein tyrosine phosphorylation events, phospholipase C (PLC)-γ1 is recruited to the PM and activated. The PLC-γ1 then hydrolyzes PI(4,5)P2 into DAG and IP3. The latter triggers the release of internal Ca^2+^ stores, causing small [Ca^2+^]_i_ elevation that induces the Ca^2+^ influx across the plasma membrane through Calcium Release-Activated Channels (CRAC). We focused our analyses on the store release rather than the influx step since several earlier studies have documented the structural and functional of impacts of PI(4,5)P2 on CRAC^23,36^. We therefore conducted the experiments in the presence of 2-APB, a specific CRAC inhibitor. The reason that we used a CRAC inhibitor rather than external Ca^2+^ chelation to block the Ca^2+^ influx was because that depletion of external Ca^2+^ was not compatible with our analyses of the TCR-mediated Ca^2+^ store release upon engagement with pMHC inasmuch as it impairs the attachment of the APCs to the substrate in our microscopy assay^37^ and also presumably affects the cell-cell adhesion between T cells and APCs^38^.

We found MCID #J cells stimulated with different doses of HEL48-63 peptide resulted in higher Ca^2+^ store release compared to WT cells (Fig. 2e). In addition, at low concentrations of loaded peptides, there also was an increase of in the percentage of the responding cells (Figure S5A). The enhanced Ca^2+^ store release stimulated by antigen was also observed in 3A9m MCID, but not MCIDi cells (Figure S5B). On the other hand, direct stimulation of Ca^2+^ store release with thapsigargin, an inhibitor of sarco-endoplasmic reticulum Ca^2+^-ATPases showed that MCID expression did not modify the Ca^2+^ store content (Figure S6B). Furthermore, since the strong reduction of PI(4,5)P2 level upon MCID expression might well affect unspecifically the production of IP3 that elicits the Ca^2+^ store release, the increased TCR-stimulated Ca^2+^ store release observed in the MCID-expressing 3A9m cells could in fact represent an underestimation of the augmented early TCR signaling in these cells. Altogether, these data are in agreement with our biochemical analysis showing that MCID expression leads to the enhanced TCR signaling initiation upon engagement with pMHC.

### The MCID expression causes modified TCR lateral diffusion at the PM

**Figure 3:**
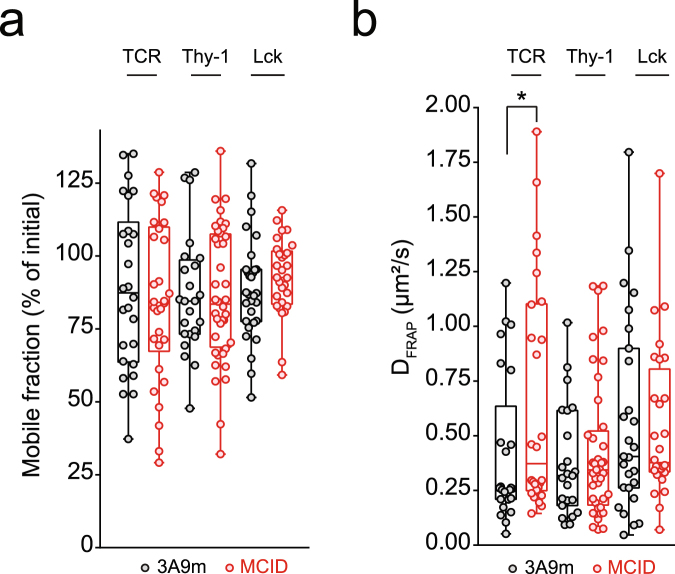
The MCID expression causes modified TCR lateral diffusion at the PM. Spot FRAP performed on 3A9m WT or MCID cells labelled with Alexa-488 conjugated Fab mAbs specific for TCR (F23.2) or Thy-1 (H194.92), or transfected with Lck::EGFP. Mobile fraction (**a**) and diffusion coefficient (**b**) for each molecule are represented by a box showing the interquartile range and whiskers extends to min and max values (n ≥ 25). *p = 0.0436 (two tailed Mann-Whitney test).

That the PI(4,5)P2 dephosphorylation heightened both the basal and pMHC-stimulated TCR activation led us to consider the role of PIs on the molecular interactions and dynamics of the TCR/CD3 complex at the PM, even prior the ligand engagement. We therefore performed spot fluorescence recovery after photobleaching (FRAP) analysis to measure the PM lateral diffusion of TCR that was surface labeled with an Alexa-488-conjugated monovalent Fab mAb. Our analysis showed that hydrolysis of PI(4,5)P2 by MCID resulted in an significant increase of the TCR diffusion rate, while not affecting the receptor mobile fraction (M_f_ ∼ 85%) (Fig. 3a,b). Indeed, the receptor’s diffusion coefficient D was 0.40 ± 0.07 µm².s^−1^ in WT and 0.65 ± 0.10 µm².s^−1^ in 3 A9m MCID cells, respectively. Moreover, this increase appeared to be selective for the TCR, since neither of the cell surface proteins Thy-1 nor an Lck:EGFP chimera exhibited modified diffusion in 3A9m MCID cells (Fig. 3a,b).

### The MCID expression alters CD3 cytoplasmic domain interactions with the PM inner leaflet

In light of our results on the involvement of PIs in the regulation of TCR membrane dynamics and signaling properties, we decided to examine their role in the regulation of the CD3 cytoplasmic domain (CD3CD) interactions with the PM. It has been recently reported that the cytoplasmic domain of CD3ε and CD3ζ contain clusters of positively charged basic amino acids, called the BRS^9,12,39,40^. The BRS was shown to bind to different anionic phospholipids including PI(4,5)P2 *in vitro*^39,40^, and its interactions with the PM internal surface were proposed to make the ITAMs in the CD3CDs unavailable for phosphorylation by Lck. As results from previous studies suggested that TCR activation upon ligand engagement involves a conformational change of the CD3CDs (in particular CD3εCD), making the ITAM accessible for phosphorylation^7,41^, the regulation of CD3CD interactions with the PM inner leaflet has naturally elicited a special interest in the context of the TCR triggering mechanism^16,42^.

**Figure 4:**
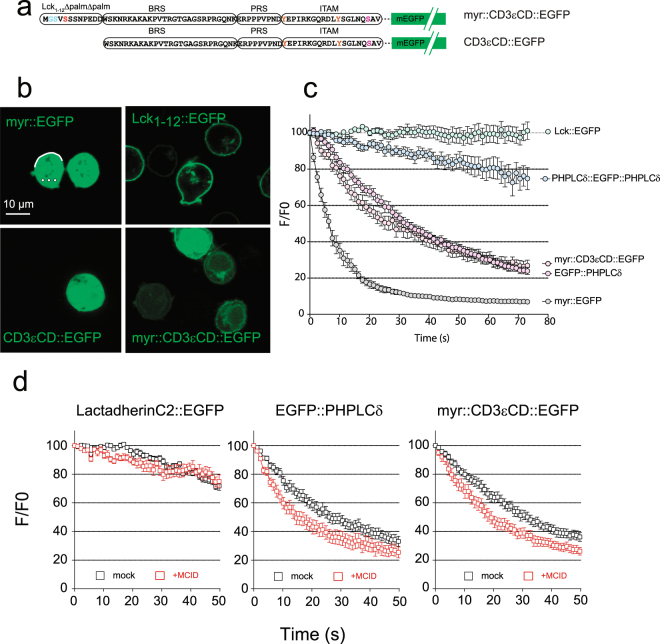
The MCID expression alters the association of CD3εCD with the PM inner leaflet in living T cells. (**a**) Schematics of CD3εCD::EGFP and myr::CD3εCD::EGFP. The CD3εCD consists of a BRS, a PRS and a ITAM segment. Myr corresponds to the first 12 amino-acids of Lck with the two palmitoylation sites (CXC) mutated into serine residus (SXS) to retain only the myristoylation site. (**b**) and (**c**) The subcellular localization of CD3εCD::EGFP, myr::CD3εCD::EGFP, myr::EGFP, and Lck_1–12_::EGFP in the JA16 Jurkat cells analysed by confocal microscopy (scale bar, 10 µm). FLIP measurements were performed by repetitive illumination of 3 intracellular circular ROI (white circles), and the membrane-cytosol exchange was monitored by recording the membrane fluorescence intenity at the opposite membrane orientation (white line) as illustrated in the “myr::EGFP” image panel, for Lck::EGFP, PH-PLCδ::EGFP::PH-PLCδ, EGFP::PH-PLCδ, myr::CD3εCD::EGFP and myr::EGFP in JA16 cells. Relative fluorescence intensities were normalized to the initial value (F/F0) (mean ± SEM of the percentage, n = 7). (**d**) FLIP measurements of the membrane-cytosol exchange of different EGFP-tagged proteins such as myr::CD3εCD::EGFP, EGFP::PH-PLCδ and Lactadherin::EGFP transfected into Jurkat JA16 cells, co-expressed (red squares) or not (black squares) with MCID.

We chose to analyze the regulation by PIs of the CD3εCD interaction with the PM inner leaflet because of it’s central functional role of CD3ε in TCR triggering mechanism^1,2^. For this, we initially transfected a construct encoding for CD3εCD conjugated with the green fluorescent protein (CD3εCD::EGFP) in Jurkat cells. However, the encoded chimeric molecule was unable to accumulate at the PM (Fig. 4a,b), indicating that its binding affinity to the PM was insufficient to yield a stable association. To facilitate “visualizing” the PM binding of the chimera, we added a myristoylation signal sequence derived from Lck_1–12_ at its the N-terminus (designed as myr::CD3εCD::EGFP). The myristic acid constitutes a weak membrane association factor that by itself did not result in detectable PM accumulation of the chimera (Fig. 4b). However, the addition of CD3εCD enabled a discernable accumulation of the chimera to the PM, indicative of the CD3εCD interaction with this cellular compartment. We then measured this interaction semi-quantitatively using the fluorescence loss in photobleaching (FLIP) technique. In this analysis, the cellular cytosolic regions were exposed to repetitive bleaching, and the molecular exchange between the PM and the cytosol was monitored in real time (Fig. 4c). The loss of the fluorescence signal upon photobleaching at the PM was found to be rapid for myr::CD3εCD::EGFP, indicating a highly dynamic interaction. This interaction was in fact similar to that for PH-PLCδ::EGFP (an established PI(4,5)P2 specific probe), stronger than that for myr::EGFP but weaker than that for 2xPH-PLCδ::EGFP or Lck_1–12_::EGFP (Fig. 4c).

The co-transfection of myr::CD3εCD::EGFP with MCID provoked a shift in its FLIP curve, indicating an increased PM dissociation rate of the chimera. The MCID expression caused a similar increase of the PM dissociation rate for PH-PLCδ::EGFP. The control experiment showed that if the PM association of these two chimeras was sensitive to PI(4,5)P2 dephosphorylation, this was not the case for LactC2::EGFP that contains the C2 domain of lactadherin, known to bind specifically to phosphatidylserine (Fig. 4d). Thus, our results showed that the MCID expression specifically alters the association of CD3εCD with the PM inner leaflet in living cells.

### The MCID expression impairs the CD3ε BRS interaction with the PM inner leaflet

**Figure 5:**
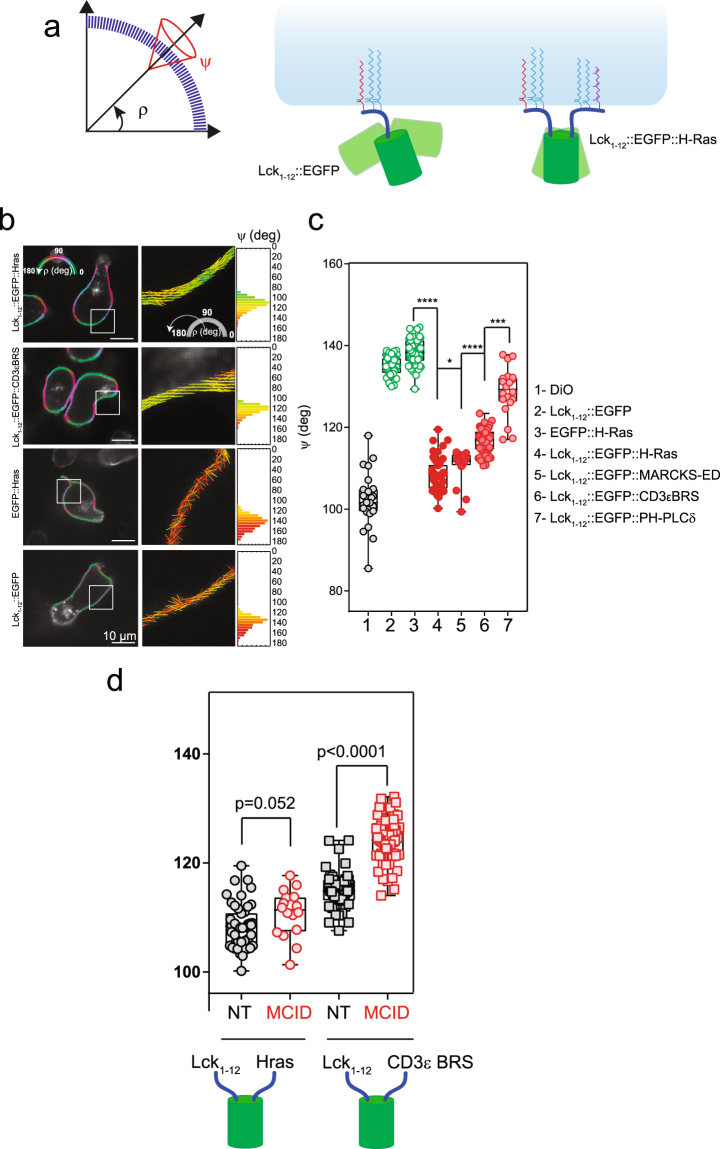
The MCID expression impairs CD3ε BRS association with PM. (**a**) Left panel: schematics of the averaged orientation (ρ) and order (ψ) parameters measured with EPRFM. Right panel: illustration of the impact of the membrane anchores (Lck_1–12_ and HrasCterm, respectively) on ρ and ψ for the chimeras. (**b**) Left panels: polarimetric images of Lck_1–12_::EGFP::Hras, Lck_1–12_::EGFP::CD3εBRS, Lck_1–12_::EGFP and EGFP::Hras expressed in 3A9m cells as determined with EPRFM. Color coded pixels referring to the average ρ angle are superimposed on the grayscale fluorescence image. Middle panels: cropped pictures from the corresponding white squares in the left panel where the averaged ρ angles are indicated by the orientation of the sticks, while the averaged ψ angles define the orders by the color code, and both parameters were determined at the sub pixel level. Right panels: the frequency histogram of the ψ value for each pixel of the whole image (n > 10000 pixels). Scale bar: 10 µm. (**c**) Statistical analysis (box and whiskers with min to max values) of the ψ value for each fluorescent chimera in each individual cell (n > 30). ****p < 0.0001, ***p < 0.001, **p < 0.01, *p < 0.05 (Mann-Whitney test). (**d**) Statistical analysis (box and whiskers with min to max values) of the ψ value for Lck_1–12_::EGFP::Hras and Lck_1–12_::EGFP::CD3εBRS in each individual 3A9m cell (n > 30), with or without MCID co-expression. Exact P-values are calculated by GraphPad Prism (Mann-Whitney test).

The above finding prompted us to examine the involvement of PIs to the interaction of CD3ε BRS with the PM inner leaflet. We though that it would be interesting to study the association of CD3ε BRS with the PM when it is already attached to the membrane via another anchor, to mimic the *in vivo* condition in living cells. For this, we used a method based on excitation-polarization-resolved fluorescence microscopy (EPRFM) which enabled us to determine the molecular orientation dynamics of fluorescent probes associated to the PM^43^. Using the EPRFM data, we could calculate the average orientation ρ of the fluorescent molecule distribution, as well as its molecular disorder (angular constraint angle) ψ with respect to the membrane within a given coordinate system (Fig. 5a).

We first performed EPRFM studies on two types of control constructs. The first was Lck_1–12_::EGFP::Hras, consisting of EGFP rimed by Lck_1–12_ (harboring one myristate acid and two palmitic acids) and Hras C-terminus peptide (harboring two palmitic acids and one farnesyl group). The second included Lck_1–12_::EGFP and EGFP::Hras, respectively. EPRFM analysis showed that for Lck_1–12_::EGFP::Hras, the mean orientation ρ of the transition dipole moment of the chromophore within the GFP was parallel to the PM, which was expected when a construct was stably double anchored to the PM. In contrast, for Lck_1–12_::EGFP or EGFP::Hras, ρ is perpendicular to the PM, indicating that when anchored only at one side, the GFP adopted a very different orientation (being perpendicular to the one with anchors on both sides) (Fig. 5b,c). Moreover, and as expected, the values of ψ depicted a greater orientational degree of freedom for Lck_1–12_::EGFP (ψ = 135.0 ± 2.2°) than Lck_1–12_::EGFP::Hras (ψ = 108.4 ± 4.0°). In fact, ψ for Lck_1–12_::EGFP::Hras is close to that for DiO (Fig. 5c), a membrane-bound fluorescent dye that should anchor to the cell membrane in a highly fixed orientation and reporting the order of the membrane surface topology^44^.

Next, we examined the construct consisting of Lck_1–12_::EGFP::CD3εBRS using EPRFM. We postulated that if the BRS of this chimera interacts with the PM inner leaflet, the behavior of the chimera as determined by EPRFM should be intermediate between the two controls analyzed above. Indeed, we found that for Lck_1–12_::EGFP::CD3εBRS ρ was parallel to the PM, similar to Lck_1–12_::EGFP::Hras. On the other hand, the value of ψ for Lck_1–12_::EGFP::CD3εBRS was higher (ψ = 116.6 ± 3.0°) than Lck_1–12_::EGFP::Hras, but significantly lower than Lck_1–12_::EGFP (Fig. 5b,c). We further observed that the ψ value of Lck_1–12_::EGFP::CD3εBRS was in the same range than that obtained for Lck_1–12_::EGFP::MARKS-ED and Lck_1–12_::EGFP::PH-PLCδ (Fig. 5c). MARKS-ED and PH-PLCδ are two protein domains with high specificity for PI(4,5)P2^17^. We observed that the ψ value found for Lck_1–12_::EGFP::MARKS-ED was lower than for PH-PLCδ, and this was consistent with the *in vitro* affinity of PI(4,5)P2 to MARKS-ED and PH-PLCδ. In conclusion, the EPRFM studies have clearly confirmed the ability of CD3ε BRS to dynamically bind to PM internal surface when it is part of a membrane protein.

We then showed that the expression of MCID significantly increased the ψ value for Lck_1–12_::EGFP::CD3εBRS (ψ = 124.2 ± 4.24°), demonstrating that the dephosphorylation of PI(4,5)P2 resulted in decreased PM binding of CD3ε BRS. The impact of the lipid phosphatase to CD3εBRS containing construct was specific as the ψ value for Lck_1–12_::EGFP::Hras was unchanged under the same experimental conditions (Fig. 5d).

### The analysis of dynamics of membrane-anchored CD3εCD chimeras at the PM

**Figure 6:**
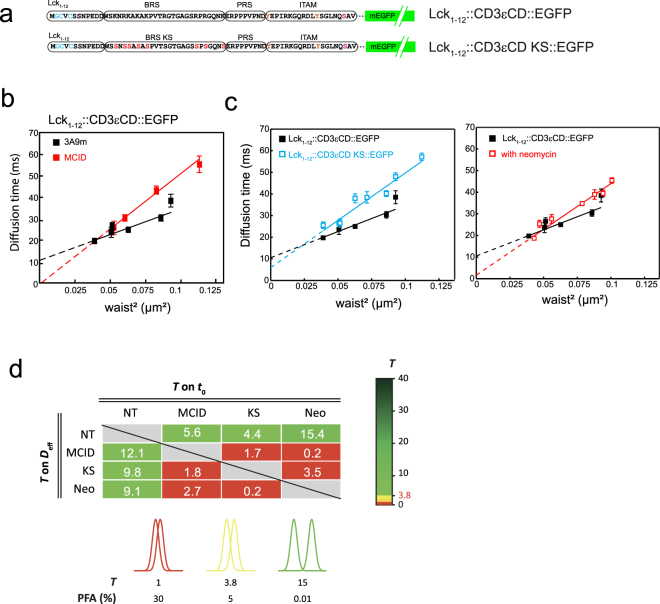
svFCS analysis of membrane-anchored CD3εCD chimeras at the PM. (**a**) Schematics of Lck_1–12_::CD3εCD::EGFP and Lck_1–12_::CD3εCD::KS::EGFP. In the latter constructs, all the lysine residues in the BRS of CD3εCD were mutated into serine residues. (**b**) Comparision of the FCS diffusion laws established for Lck_1–12_::CD3εCD::EGFP expressed in the 3A9m T cells, in the presence (red squares) or absence of MCID co-expression (black squares). The measurements were performed at 37 °C (see Methods). (**c**) Left panel: comparison of the FCS diffusion law established for Lck_1–12_::CD3εCD::EGFP (black squares) and for Lck_1–12_::CD3εCD KS::EGFP (blue squares), expressed in 3A9m T cells. Right panel: comparison of the FCS diffusion law established for Lck_1–12_::CD3εCD::EGFP expressed in 3A9m T cells, in the presence (red squares) or absence of 2 mM neomycin pre-treatment (black squares). (**d**) Statistical comparison of *t*_0_ and *D*_eff_ determined in the svFCS experiments. $T$ values of statistical significance were calculated by pairwise comparison of *t*_0_ and *D*_eff_ and color-coded as a function of the PFA threshold (see Methods for details).

The binding of CD3 BRS to the PM could also impact the membrane lateral dynamics of the entire molecule possessing such a cytoplasmic domain. To test this, we examined the membrane dynamics of a GFP-conjugated CD3εCD construct which was anchored to the PM via Lck_1–12_ using spot-variation fluorescence correlation spectroscopy (svFCS). svFCS is a unique approach that allows the characterization of PM dynamics with high spatio-temporal resolution and is able to identify molecular lateral diffusion confinements efficiently. Lck_1–12_::EGFP was previously shown dynamic partitioning/assembling into sphingolipid and cholesterol-dependent membrane nanodomains (also known as raft nanodomains)^45,46^. Therefore, we were interested to investigate CD3εCD chimeric molecules attached to the PM via Lck_1–12,_ as raft nanodomains have been previously proposed to be a functionally important environment for TCR triggering, and especially in view of the recent report on the direct binding of CD3ε BRS to Lck. The svFCS analysis showed that Lck_1–12_::CD3εCD::EGFP (Fig. 6a) expressed in 3A9m T cells displayed a high mobility (*D*_*eff*_ = 1.04 ± 0.13 µm²/s), with a positive confinement index value (*t*_0_ = 11.43 ± 1.99 ms) that is indicative of its partitioning into membrane nanodomains/nanoassemblies. Interestingly, upon expression of MCID in the same cells, there was a reduction of mobility (*D*_*eff*_ = 0.48 ± 0.06 µm²/s), accompanied by a strong drop in the confinement (*t*_0_ = 0.66 ± 4.69 ms) of the chimera, indicating that PI(4,5)P2 contributes to Lck_1–12_::CD3εCD::EGFP nanodomain partitioning (Fig. 6b). A similar change was observed (*D*_*eff*_ = 0.57 ± 0.03 µm²/s; *t*_0_ = 5.91 ± 1.74 ms) when all lysines in the BRS were mutated into serine, which gave rise to Lck_1–12_::CD3εCD-KS::EGFP (Fig. 6a,c). As the KS mutation was previously shown to abolish BRS interactions with anionic lipids in the membrane, this indicated that these BRS-lipid interactions could promote the nanoconfinement of the chimera, and suggested that the impact of PI(4,5)P2 on these interactions possibly accounts for its contribution to the membrane dynamics of the modified chimera. 3A9m T cells treated with neomycin produced a similar effect on the Lck_1–12_::CD3εCD::EGFP lateral diffusion (*D*_*eff*_ = 0.59 ± 0.03 µm²/s; *t*_0_ = 1.64 ± 1.50 ms) as the KS mutation (Fig. 6c). Neomycin is an aminoglycoside that specifically and strongly interacts with the poly-PIs, especially PI(4,5)P2^47^, and blocks ionic interactions of PI(4,5)P2 with endogenous proteins *in vivo*^48,49^. These data thus were consistent with those upon MCID expression and supported that the PI(4,5)P2-based ionic molecular interactions contribute to the BRS-promoted Lck_1–12_::CD3εCD::EGFP lateral nanoconfinement at the PM.

## Discussion

The PIs, in particular PI(4,5)P2, play multiple and versatile roles in the activities of numerous proteins, especially those localized in the PM, as exemplified by large number of ion channels whose activities are tightly regulated by its binding to PI(4,5)P2^18,19^. In addition, the interaction of PI(4,5)P2 with many membrane receptors modulates their function^17^. In this study, we were interested in the role of PI(4,5)P2 in TCR triggering, a process that has remained poorly understood despite several decades of extensive studies and accumulated information on its protein participants. The lack of comprehension could be at least in part due to the scarce information currently available on the interactions of TCR/CD3 complex with lipids in the PM and the real difficulty to investigate these interactions in the physiological contexts.

To examine the contribution of PI(4,5)P2 in the TCR triggering mechanism, we expressed MCID, a mCherry-conjugated Inp54p construct anchored to the membrane via Lck_1–12_ with a stable association to the PM. The exact same strategy has previously been used for the PM targeting of the functional protein units, which revealed various key aspects of the TCR triggering mechanism^50,51^. The MCID expression brought about a substantial hydrolysis of PM PI(4,5)P2 without significant modification of F-actin cortex. However, the TCR activation and signaling initiation upon pMHC engagment was noticeably augmented in these cells. Of note, a previous study using the Inp54p attached to the PM via a similar anchor to MCID (i.e., Lck_1–10_) in Jurkat cells did not reveal any significant impact of the phosphatase on TCR signaling^22^, despite cytoskeletal alterations induced by the construct. The precise reason for the apparent discrepancy with our present work is currently not clear. It should be mentioned that in that study, the total PI(4,5)P2 level did not change upon the phosphatase expression, and only a small reduction (10%) for the PI(4,5)P2 present in the fraction co-purified with the detergent-resistant membranes was observed. Second, the TCR signaling was examined only with the stimulation made with soluble anti-CD3 mAb but not with pMHC presented by the APC^22^. In fact, in our study, MCID expression showed no effects on the soluble anti-CD3 mAb-mediated signaling. Finally, since Jurkat cells display high basal level of PI(3,4,5)P3 due to constitutive PTEN and SHIP-1 lipid phosphatase deletion^52,53^, some observed cytoskeletal changes might be linked to the modification of PI(3,4,5)P3 level upon Inp54p expression.

That MCID expression only increased the TCR triggering stimulated with pMHC, but not with soluble anti-CD3 mAb was not particularly surprising. Recent studies revealing that the activation of TCR upon the dynamic 2D binding with pMHC in physiological conditions is likely to occur through mechanisms that are quite different from those observed when the receptor is stimulated by antibody crosslinking^54,55^. On the other hand, the differential effects of MCID expression on the pMHC- and soluble anti-CD3-elicited TCR activation suggests that these effects are not due to TCR-unrelated alterations such as a higher Lck activity^56^ or positive feedback effects from down-stream signaling events such as a stronger calcium influx^10^. Additionally, in our opinion, the effects of the phosphatase on TCR activation were not due to diminished cell contacts, owing to results from a previous study that showed that the reduction of PI(4,5)P2 levels in T cells promotes rather than inhibits the contacts between T cells and APCs^57^, which could be a result of decreased T cell membrane rigidity^58^. These considerations, together with the augmented basal phosphorylation of CD3 observed in MCID-expressing cells, led us to favor the hypothesis that the PI(4,5)P2 dephosphorylation results in changes in the PM molecular interactions, which can significantly facilitate the availability/interactions of CD3 with the constitutively activated Lck for CD3 ITAM phophorylation. This was in fact reminescent to the conformation change model of the TCR activation, proposing that membrane-association of CD3CD critically controls the phosphorylation of the TCR/CD3 complex^9,11,12^. The proposition that MCID expression could provoke a higher Lck accessibility/interaction for CD3 was also consistent with our FRAP analysis revealing that the same expression leaded to a specific increase of the TCR/CD3 lateral mobility in the PM.

The CD3ε and CD3ζ encompass BRS in their cytoplasmic domain and have been proposed to chiefly control the CD3CD association with the PM inner leaflet upon interactions with negatively charged lipids localized at this place. It was observed that the CD3εCD^39^ (as well as CD3ζCD)^40^ bound to different anionic lipids including various PIs *in vitro* via their BRS. These studies also showed that when presented in the form of lipid arrays, the phosphatidylinositol monophosphates, PI3P, PI4P and PI5P, displayed higher relative binding affinities to BRS than phosphatidylinositol bisphosphates and trisphosphates. However, when incorporated into artificial lipid bilayer membranes, the binding of PI(4,5)P2 to BRS was increased drastically over the phosphatidylinositol monophosphates and emerged as the ligand with the highest relative affinity among the tested PIs^39,40^. These observations, alongside the fact that PI(4,5)P2 is one of the most abundant PIs in the PM suggest that PI(4,5)P2 represents a physiological interactor for CD3εCD in the PM inner leaflet. The results of our present study conducted *in vivo* further substantiate this proposal. Indeed, using various state-of-the-art biophotonic approaches, we have shown in living cells that the hydrolysis of PI(4,5)P2 by MCID resulted in the unbinding of CD3εCD and CD3ε BRS from the PM inner leaflet. Although an increase in PM PI4P levels cannot be excluded in these experiments due to limitations of the immunostaining method, we believe that it was the decrease in the PM level of PI(4,5)P2, rather than a possible increase in that of PI4P, that is responsible for the dissociation of CD3εCD and its BRS from the PM inner leaflet. Such an increase, in the event that it occurs, would in fact counterbalance the depletion of PI(4,5)P2 to induce the dissociation of CD3εCD from BRS PM due to the binding of PI4P to BRS. Consistent with the idea that the depletion of PI(4,5)P2 being the cause of the disruption of BRS-mediated PM association of CD3εCD, we observed that the sequestration of PI(4,5)P2 by neomycin produced an inhibitory effect that was quantitatively similar to that of the MCID expression on the BRS-dependent dynamic confinement of Lck_1–12_::CD3εCD::EGFP at the PM (Fig. 6).

Beside the direct binding to BRS, PI(4,5)P2 could also contribute to CD3ε BRS-PM association by additional means, for instance via establishement of an adequate membrane electrostatic potential^51^. Indeed, it has recently been proposed that the specific molecular interactions and surface electrostatic potential may conjointly promote the CD3CD association with the PM. In addition, in a previous study based on a focal decrease of the availability at the immunological synapse, phosphatidylserine (PS) has been put forward as an anionic lipid responsible for the CD3CD binding to the PM inner leaflet^12^. Although such a functional role for PS remains controversial^51,59^, we think that PI(4,5)P2 and PS could work cooperatively^60^, possibly along with cholesterol^61^ to contribute to the assembly of the TCR-lipid nanodomains whereby CD3CD association with the PM inner leaflet is promoted.

In recent years, the model of “membrane-dissociation-of-CD3CD-for-ITAM-phosphorylation” for the TCR triggering mechanism has elicited a lot of interest. A key role of CD3ε BRS interaction with membrane anionic lipids is heavily debated^13,14^ as there has been a lack of the expected functional phenotype, *i.e*., increased ITAM phosphorylation, with the KS mutation of CD3ε BRS. It now is known that this observation presumably is because that such mutation causes both a higher accessibility and a lower affinity of CD3εCD for Lck^15^, thereby complicating the interpretation for the experiments of phosphorylation of the CD3ε with BRS KS mutation. Thus, as such, our data on the reduced levels of PI(4,5)P2 resulted in an augmentation of the basal and pMHC-stimulated CD3 phosphorylation in parallel with a significant diminution of the BRS-dependent CD3εCD association with the PM support a crucial role of the BRS-controlled CD3εCD orthogonal dynamics on the PM inner surface in the TCR triggering.

In conclusion, our study strongly suggests that PI(4,5)P2 is a key regulator of the TCR membrane dynamics and controls of TCR activation and initiation of signaling pathway. In addition, our results provide new insights on the TCR triggering mechanism, especially on the dynamic dissociation of CD3CD from the PM inner leaflet upon the receptor engagement. Moreover, the contribution of PI(4,5)P2 may not limit to the binding of CD3CD to the PM inner leaflet in regulating TCR/CD3 activation, since the membrane proximal BRS is also found in the cytoplasmic domain of other immunoreceptor on the PM of lymphocytes^62^.

## Methods

### Reagents and antibodies

2-Aminoethoxydiphenylborate (2-APB), thapsigargin, Latrunculin A, U-73122 were purchased from Calbiochem. m-3M3FBS, and neomycin were from Sigma-Aldrich. The PBX calcium assay kit, the antibodies against CD3ε (clone 145- 2C11), the F23.1 anti-TCR Vβ 8 1–3 antibody were supplied by Becton Dickinson. The FITC-conjugated anti-PI(4,5)P2 and anti-PI4P mAbs were from Echelon Biosciences. Alexa Fluor^TM^ 488-conjugated Dnase1 and Alexa Fluor^TM^-conjugated phalloidin were supplied by Life technologies (Molecular probes). The anti-Vβ8 TCR (F23.2), anti-Cβ (H57-597), anti-CD3ε (145-2C11), anti-CD4 (GK1.5), anti-CD45 (H193.16.3), anti-Thy-1 (H194.92) (10 mg/mL, final) were purified from cell culture supernatants of the hybridomas in the lab; according to standard protocols. The antibodies purchased for immunoblotting were as follows: mouse anti-phosphotyrosine (4G10; Merck Millipore); anti-Zap-70 (clone 29/Zap70, BD Biosciences); anti-phospho-Zap70 (Tyr319) (Cell Signaling). H146-968 (anti-CD3ζ) mAb was produced in the lab. All other chemical reagents were from Sigma–Aldrich. [^32^P]orthophosphate was from PerkinElmer.

### EGFP and mCherry-conjugated chimeras

All fluorescent-protein chimera were generated upon different conventional molecular biology strategies as summarized in Table S1. Both the EGFP (monomeric enhanced GFP) and mCherry are monomeric fluorescent proteins. Inserts (annealed oligos, PCR amplicons) were cloned into the pEGFP-N2 backbone (Clontech). Restriction enzymes as well as T4 DNA ligase were from New England Biolabs. PCR amplifications as overlap extension PCR^63^ were performed with Pfu ultra DNA polymerase from Agilent. In some cases, PCR fragments were inserted into linearized vector upon *in-fusion* methods (Clontech), in presence of cloning enhancer, according to the manufacturer guidelines. Site directed mutagenesis were performed using the QuikChange II XL Site-Directed Mutagenesis Kit (Agilent). All constructs were verified by sequencing (MWG Biotech).

### Fluorescent monovalent Fragment Antibodies (Fabs)

Specific mAbs were purified on Protein-G sepharose affinity chromatography column (GE HeathCare) and eluted with HCl-Glycine 0.1 M pH 2.8 in 1 M Tris-HCl, pH 9 and further dialyzed against PBS. Fabs were generated by papain or fucin digestion (according to their isotype) using 50 μg of enzyme per 1 mg of mAb in PBS, 0.01 M EDTA, 0.01 M cysteine, pH 7 at 37 °C. Digestion was stopped with addition of 0.03 M iodo-acetamide and dialyzed against PBS. Fabs were labeled with Alexa 488 dyes (Invitrogen) according to manufacturer’s instructions. Dye/protein ratios were adjusted to 1–3 moles of Alexa dye per mole of Fab.

### Cells

Jurkat cells (JA16 clone) were cultured in RPMI 10% FCS, 1 mM sodium pyruvate and 10 mM Hepes. The 3A9 CD4^+^ T cell hybridoma displays a TCR specific for hen egg lysozyme peptide (HEL) bound to MHC II I-A^k^ molecules^27^. 3A9m sub-line was established according to its high TCR expression. These cells were cultured in RPMI medium supplemented with 5% FCS, 1 mM sodium pyruvate and 10 mM Hepes (complete RPMI).

Stable cell lines were obtained by electroporation using Amaxa nucleofactor (Lonza) according to manufacturer protocols (3A9m protocol B024 solution V) and selected upon resistance to puromycin (1.3 μg/ml 3A9m or 5 μg/ml COS-7) conferred by the co-transfection of the pMSCV-puro plasmid (Clontech) (ratio 1/25 regarding to expression plasmid). Positive subpopulations were isolated by cell sorting (FACSARIA, Becton Dickinson) coupled to auto-cloning in 96-well plates (5 positive cells/well). For the 3A9m transfectants, cell sorting was carried out according to good mCherry and TCR expression levels, which allowed establishing 3A9m MCID cells. The individual clones were established subsequently in a second round of cell sorting/auto-cloning (one positive cell/well). For the cells that co-expressed MCID and EGFP-conjugated protein probes, they were obtained upon transient expression of MCID following nucleofection of 3A9m cells stably expressing various EGFP-conjugated protein probes (obtained by cell sorting similarly as for 3A9m MCID cells).

Antigen-presenting cells (APCs) were generated by stably transfecting COS-7 cells (Amaxa, V solution, A024) with plasmid cDNAs coding for the α chain of MHC II and the β chain of MHC II I-A^K^ alone or covalently fused to a peptide derived from HEL (HEL 48–63)^64^ (provided by D.A. Vignali). Cells were sorted according to their positivity to surface labeling by C4H3 antibodies (Facsvantage, Becton Dickinson). COS-7 cells were cultured in DMEM medium with 5% FCS, 1 mM sodium pyruvate and 10 mM Hepes. APC monolayers were generated by seeding 5.5 × 10^4^ cells onto poly-L-Lysine-coated 8-well Lab-Tek chamber (Nunc).

### SDS-PAGE, immunoblotting and quantification

The SDS-PAGE and immunoblotting were conducted as previously described^65^. Briefly, cells were lysed at 95 °C for 5 min in sample loading buffer (2% SDS, 60 mM Tris, pH 6.8). After ultracentrifugation (85,000 rpm, 20 min), supernatant was recovered and the proteins were resolved by SDS-PAGE and transferred onto the PVDF membrane. The proteins were then blotted with specific antibodies followed by chemiluminescence detection (ECL, Amersham Pharmacia biotech). For the quantification of signals in immunoblots, we measured the chemiluminescence signals using a Bioimage analyzer LAS-1000 and its dedicated software (MultiGauge, Fujifilm).

### Flow cytometry

Cells were analyzed on a LSR II flow cytometer (Becton Dickinson) using the FACSDiva software. Data analysis was performed with FlowJo (Tree star) and the median intensity of fluorescence was plotted vs time after exclusion of dead cells and cell debris.

### F- and G-actin analysis by flow cytometry

Paraformaldehyde-treated cells (4% PFA, 15 min, RT) were subsequently incubated in a solution containing PBS, 10% FCS, 0.05% Saponin permeabilization solution in the presence of 20 µM Alexa Fluor^TM^ 546-Phalloidin and 5 µg/ml of Alexa Fluor^TM^ 488 DNASE I (Life technologies).

### F-actin labeling for super-resolution microscopic analysis

Actin meshwork architecture analysis were performed on Alexa Fluor^TM^ 647 phalloidin labelled cells seeded onto anti-CD45 mAb (H193.16.3) coated slides. Fixation and permeabilization (0.3% glutaraldehyde and 0.25% Triton X-100 then 2% glutaraldehyde), quenching (0.1% NaBH_4_) and labelling (Alexa Fluor^TM^ 647 phalloidin 0.5 µM) were performed according to^66^.

### Cell treatment with PI3K inhibitor

The cells seeded onto anti-CD45 mAb coated slides were treated with 20 µM PI3K inhibitor LY294002 for 30 min at 37 °C before submitting to super-resolution microscopic analysis of actin cortex.

### PM PI(4,5)P2 and PI4P staining

The PM PI(4,5)P_2_ and PI4P staining were performed according to^28^. Cells in culture medium were immediately fixed by the addition of PBS-diluted fixative to a final concentration of 4% paraformaldehyde and 0.2% glutaraldehyde, for 15 min at 30 °C. Fixed cells were transferred on ice, and all subsequent steps were performed on ice with pre-chilled solutions. Cells were washed twice in ice-cold PBS. Cells were then blocked in a blocking buffer containing PBS and 0.1 M Glycine for 45 min, washed twice with PBS and then permeabilized with 5% normal goat serum (NGS) with digitonin (10 µg/ml final) in PBS for an additional 45 min. Cells were washed twice in 5% NGS in PBS, before being labelled overnight with FITC-conjugated antibodies diluted in PBS containing 5% NGS. After two washes in PBS, cells were post-fixed in 2% PFA in PBS for 10 min on ice. Labelling was evaluated either using flow cytometer or by confocal microscopy.

### Cell preparation and lipid extraction

For metabolic labeling, cells were cultured in phosphate-free RPMI medium in the presence of 1 mCi/mL [^32^Pi]-orthophosphate during 8 hours, lipids were then extracted by the acidic Bligh and Dyer procedure and their phosphoinositide content was analyzed as described previously^67^. Briefly, lipids extracted from the different cells were resolved by thin layer chromatography using a solvent of CHCl_3_/CH_3_OH/NH_4_OH 4,3 N (90/70/20, v/v) on silica-coated glass plates pre-activated for 30 min at 70 °C after migration in a solution of 50% CH_3_OH, 1% potassium oxalate, 2 mM EDTA. The migration of authentic standards visualized by iodine vapours allowed the localization of ^32^P-PI4P and ^32^P-PI(4,5)P_2_ in the different samples. PI4P and PI(4,5)P_2_ were then scrapped off and extracted by the acidic Bligh and Dyer procedure, deacylated and futher analyzed by HPLC^67^.

### Confocal and super-resolution microscopy

Video confocal imaging were mostly performed on a Zeiss LSM 510 Meta confocal microscope equipped with a 30 mW argon laser (25% output,1% AOTF). Pictures were taken with a C-Apochromat 406/1.2 water immersion objective, using the 488 nm line of the argon laser, HFT UV/488 dichroic mirror and a 505 nm long pass filter at 37 °C, maintained using a hot plate. Time-lapse movies were composed of 300 images (512 × 512 pixels; 8 bit; 225 µmx225 µm; pinhole set to 3 airy units) taken every 7 seconds. Additional observations were performed on an Ultraview VoX Perkin Elmer spinning disk confocal microscope, equipped with two synchronous EMCCD cameras. The actin cytoskeleton architecture was imaged on a Zeiss LSM 880 confocal microscope equipped with a sensitive and super-resolved Fast-AiryScan detector. Samples were illuminated by a 633 nm laser through a Plan-Apochromat 63X/1.4 NA oil immersion objective. 16 bits images corresponding to 12 µm z-stack satisfying the Nyquist criterion were processed by the inbuilt AiryScan fonction embedded in the acquisition/analysis software (Zen, Zeiss, Germany).

### FLIP experiments

FLIP imaging was adapted from^45^. Briefly, recordings were performed at 37 °C on a Zeiss LSM 510 confocal microscope using the 488 nm line of a 25 mW argon laser beam, a Zeiss plan-Apochromat 63×, NA = 1.4 oil immersion objective and the filter set supplied by the manufacturer. Cells were repeatedly photo-bleached within the cytosolic regions of interest (ROI) (Ø = 1.24 µm). Between the bleaching periods, the cells were imaged with low light intensity. We recorded one image every 1.13 s at 1% laser transmission, alternating with 20 iterations photo-bleaching illuminations (~1.8 s) at 100% laser transmission. Plasma membrane fluorescence decay was measured in a 5 pixel-width segmented line. Average fluorescence in the region was quantified with a homemade ImageJ macro (available on request) and plotted versus time. Graphs showed the average fluorescence measurement of at least four individual recordings ± s.e.m.

### Measurement of T cell cytosolic calcium concentration (and velocity)

The measurement of CD4^+^ T cell cytosolic calcium was performed according to a newly developed method in the laboratory^37^. In brief, 5 × 10^4^ T cells per well were plated in 96-well plates in 100 µL of complete medium. Cells were loaded with BD PBX dye diluted in 1X dye loading solution (according to manufacturer’s instructions) at 1/1000^e^ (~1 µM) for 3A9m cells. 100 µL of this solution were dispatched to each well before incubation at 37 °C for 1 hour in the dark. Cells were then washed twice in Hank’s balanced salt solution (HBSS) Hepes buffer containing 1 mM calcium and resuspended in the same medium. Five wells were pooled (2.5 × 10^5^ cells) and analyzed either by flow cytometry or microscopy. 2-APB was added just before the recordings. Indifferently, BD PBX loaded T cell calcium responses were evaluated by time lapse recording either by a flow cytometer or videomicroscopy. Cells were analyzed on a LSR II flow cytometer (Becton Dickinson) using the FACSDiva software. PBX calcium indicator was observed over time on the FL1 channel with an excitation by an Argon laser 488 nm and a 530/30 nm emission filter at 37 °C, maintained using a water bath. Data analysis was performed with FlowJo software and the median intensity of fluorescence was plotted vs. time after exclusion of dead cells and cell debris. Time-lapse movies of the cells, composed of 300 images taken every 7 seconds, were performed on a Zeiss LSM 510 Meta or 780 confocal microscope equipped with a C-Apochromat 40X/1.2 water immersion objective as well as an argon laser with a 488 nm dichroic and a 505 nm long pass filter. During the movie recording, cells were kept at 37 °C using a hot plate. Calcium response intensity parameters as well as velocity and cell shape were determined by MAAACS as described^37^. Specific calcium response amplitude was determined upon specific thresholds (depending on experimental stimuli) set according to our previous study. Percentage of responding cells, the average response amplitude, the average response time fraction and the average burst duration were automatically calculated and tabulated in excel data sheets. Results were plotted as dot plots, limited to calcium responses above threshold. Data shows mean +/-std. error.

### Anti-CD3 mAb cell binding analysis

5 × 10^5^ cells were washed twice in DMEM F12 medium with 1% Nutridoma prior to flow cytometry. Cell autofluorescence was recorded for 1 min (and was considered as the baseline) prior to adding Alexa 488-conjugated anti-CD3ε mAb (5 µg/ml). Fluorescence signal was recorded for 452 additional seconds as described in^68^.

### svFCS and FRAP analysis

svFCS and spot FRAP measurements were performed on a Zeiss C-Apochromat custom apparatus as previously described^69–71^. T cells were seeded onto polylysine coated Lab-Tek slides (Nunc). All Alexa-488-Fab labellings were performed at 10 °C for 30 min prior to FRAP recordings at 37 °C in DMEM-F12 (without phenol red) supplemented with 1% Nutridoma. Overnight metabolic treatments were kept during measurements. Data were analyzed using an in-house software written in IGOR Pro (wavemetrics) and Matlab (Mathworks). The defined point spread function (namely the waist size, ranging from 200–330 nm) was preliminary calibrated according to the Rho6G diffusion coefficient (280 µm²/s in aqueous solution), the estimation of the measurement error being linear with the w² value not exceeding 0.5% even for the larger waist.

The detailed procedure for svFCS acquisition and analysis has been extensively described in^46,69–71^. Briefly, for each observation spot size, each series (between 10 to 15 series of twenty 5 second-recordings) was performed on a different low fluorescent cells (density~50 molecules/µm² evaluated from FCS measurements). For each series, the 20 recordings were selected in order to remove unexpected fluctuations visible on the intensity fluctuations (upon a unique criteria of fluorescence fluctuation around a stationary regime of the count rate) or unexpected shape of the ACF (Auto-Correlation Function). Next, the mean of the retained ACF was fitted with a 2 component model where the slow one (named τ_d_) reflects the mean residence time of molecules within the observation spot. This is repeated for 10 to 15 series, and the mean diffusion time, which is weighted in accordance to the number of kept ACF per series, is calculated. The estimation of the τ_d_ and ω^2^ values were determined using a maximum likelihood method considering a Gaussian kernel of the individual and independent recordings with their experimental standard deviations. The average τ_d_ (for each series at a defined waist) and their associated errors (square root of the variance) were plotted as a function of the ω^2^ (named Diffusion Law, DL). The accuracy of the final result was assessed by a linear regression analysis which simultaneously accounts for the estimation errors of τ_d_ and ω^2^. The estimation variances for t_0_ (y-axis intercept of the DL) and D_eff_ (effective diffusion coefficient, proportional to the inverse of the slope of the DL) according to this formula: τ_d_ = t_0_ + (1/4D_eff_)* ω^2^ were given by the Cramér-Rao bound. Each FCS diffusion law was obtained from at least 800 individual recordings.

Finally, to estimate the statistical significance between two diffusion laws, testing hypotheses were performed independently for t_0_ and D_eff_ accounting for their variances, returning a value of the probability of false alarm (PFA)^37,72^. These percentages can be considered as the probability of the chance the compared values are originated from the same statistical process. As an example a PFA of 5% indicated that there is only 5% of chance the two values are identical, which we considered as the superior limit of significant difference between two parameters. The overall statistical analysis is summarized in a 2D color-coded table (t_0_ vs D_eff_).

The spot FRAP measurements were performed at 37 °C using the same setup except that the bleaching step was performed by illuminating the sample for 1 ms at 1 mW. Both, the pre- and post-bleaching steps were performed at 2 µW but for a duration of 500 ms and 2,000 ms, respectively. All individual measurements were carried out on a minimum of 20 different cells. The data were fitted by equation (3) as previously described^71,73^:

1 $$\frac{F(t)}{{F}_{p}}=\alpha \mathop{\sum }\limits_{n=0}^{\infty }[(\frac{{(-K)}^{n}}{n!})(\frac{1}{1+n(1+2t/{\tau }_{d})})]+(1-\alpha )\frac{{F}_{0}}{{F}_{p}}$$

where α represents the mobile fraction, K a parameter relating to the deepness of bleaching, τ_d_ the characteristic recovery time, and F_p_ and F_0_ the fluorescence intensities prior to and immediately after the bleaching step, respectively.

### Statistical analysis

All statistical analyses were performed using GraphPad Prism 5.00. A non- parametric two-tailed unpaired Mann-Whitney test was used, unless otherwise stated.

## Electronic supplementary material

Supplementary Information

## References

[1] Smith-Garvin JE, Koretzky GA, Jordan MS (2009). T cell activation. Annu Rev Immunol.

[2] Malissen B, Bongrand P (2015). Early T cell activation: integrating biochemical, structural, and biophysical cues. Annual review of immunology.

[3] Weiss A, Littman DR (1994). Signal transduction by lymphocyte antigen receptors. Cell.

[4] Dorfman JR, Stefanova I, Yasutomo K, Germain RN (2000). CD4+ T cell survival is not directly linked to self-MHC-induced TCR signaling. Nat Immunol.

[5] Nika K (2010). Constitutively active Lck kinase in T cells drives antigen receptor signal transduction. Immunity.

[6] Krogsgaard M (2005). Agonist/endogenous peptide-MHC heterodimers drive T cell activation and sensitivity. Nature.

[7] Gil D, Schamel WW, Montoya M, Sanchez-Madrid F, Alarcon B (2002). Recruitment of Nck by CD3 epsilon reveals a ligand-induced conformational change essential for T cell receptor signaling and synapse formation. Cell.

[8] Davis SJ, van der Merwe PA (2006). The kinetic-segregation model: TCR triggering and beyond. Nature immunology.

[9] Xu C (2008). Regulation of T cell receptor activation by dynamic membrane binding of the CD3epsilon cytoplasmic tyrosine-based motif. Cell.

[10] Shi X (2013). Ca2+ regulates T-cell receptor activation by modulating the charge property of lipids. Nature.

[11] Guo X (2017). Lipid-dependent conformational dynamics underlie the functional versatility of T-cell receptor. Cell Research.

[12] Gagnon E, Schubert DA, Gordo S, Chu HH, Wucherpfennig KW (2012). Local changes in lipid environment of TCR microclusters regulate membrane binding by the CD3epsilon cytoplasmic domain. J Exp Med.

[13] Fernandes RA (2010). What controls T cell receptor phosphorylation?. Cell.

[14] Gagnon E (2010). Response multilayered control of T cell receptor phosphorylation. Cell.

[15] Li, L. *et al*. Ionic CD3− Lck interaction regulates the initiation of T-cell receptor signaling. *Proceedings of the National Academy of Sciences*, 201701990 (2017). 10.1073/pnas.1701990114 PMC5530670 28659468

[16] Brownlie RJ, Zamoyska R (2013). T cell receptor signalling networks: branched, diversified and bounded. Nature reviews. Immunology.

[17] McLaughlin S, Murray D (2005). Plasma membrane phosphoinositide organization by protein electrostatics. Nature.

[18] Di Paolo G, De Camilli P (2006). Phosphoinositides in cell regulation and membrane dynamics. Nature.

[19] Balla T, Szentpetery Z, Kim YJ (2009). Phosphoinositide signaling: new tools and insights. Physiology.

[20] Berridge MJ, Dawson R, Downes C, Heslop J, Irvine R (1983). Changes in the levels of inositol phosphates after agonist-dependent hydrolysis of membrane phosphoinositides. Biochemical Journal.

[21] Cantley LC (2002). The phosphoinositide 3-kinase pathway. Science.

[22] Johnson CM, Chichili GR, Rodgers W (2008). Compartmentalization of phosphatidylinositol 4,5-bisphosphate signaling evidenced using targeted phosphatases. J Biol Chem.

[23] Calloway N (2011). Stimulated association of STIM1 and Orai1 is regulated by the balance of PtdIns (4, 5) P2 between distinct membrane pools. J Cell Sci.

[24] Wang J, Richards DA (2012). Segregation of PIP2 and PIP3 into distinct nanoscale regions within the plasma membrane. Biol Open.

[25] Wiradjaja F (2001). The yeast inositol polyphosphate 5-phosphatase Inp54p localizes to the endoplasmic reticulum via a C-terminal hydrophobic anchoring tail: regulation of secretion from the endoplasmic reticulum. J Biol Chem.

[26] He HT, Marguet D (2008). T-cell antigen receptor triggering and lipid rafts: a matter of space and time scales. Talking Point on the involvement of lipid rafts in T-cell activation. EMBO Rep.

[27] Vignali DA, Strominger JL (1994). Amino acid residues that flank core peptide epitopes and the extracellular domains of CD4 modulate differential signaling through the T cell receptor. J Exp Med.

[28] Hammond GR, Schiavo G, Irvine RF (2009). Immunocytochemical techniques reveal multiple, distinct cellular pools of PtdIns4P and PtdIns (4, 5) P2. Biochemical Journal.

[29] Hammond GR (2012). PI4P and PI(4,5)P2 are essential but independent lipid determinants of membrane identity. Science.

[30] Tsujishita Y, Guo S, Stolz LE, York JD, Hurley JH (2001). Specificity determinants in phosphoinositide dephosphorylation: crystal structure of an archetypal inositol polyphosphate 5-phosphatase. Cell.

[31] Laux T (2000). GAP43, MARCKS, and CAP23 modulate PI(4,5)P(2) at plasmalemmal rafts, and regulate cell cortex actin dynamics through a common mechanism. J Cell Biol.

[32] Lee KH (2006). The role of receptor internalization in CD95 signaling. The EMBO journal.

[33] Hammond GR (2006). Elimination of plasma membrane phosphatidylinositol (4, 5)-bisphosphate is required for exocytosis from mast cells. Journal of cell science.

[34] Chakraborty AK, Weiss A (2014). Insights into the initiation of TCR signaling. Nat Immunol.

[35] Feske S (2007). Calcium signalling in lymphocyte activation and disease. Nat Rev Immunol.

[36] Walsh CM (2010). Role of phosphoinositides in STIM1 dynamics and store-operated calcium entry. Biochemical Journal.

[37] Salles A (2013). Barcoding T cell calcium response diversity with methods for automated and accurate analysis of cell signals (MAAACS). PLoS Comput Biol.

[38] van Kooyk Y, Weder P, Heije K, Figdor CG (1994). Extracellular Ca2+ modulates leukocyte function-associated antigen-1 cell surface distribution on T lymphocytes and consequently affects cell adhesion. J Cell Biol.

[39] Deford-Watts LM (2011). The CD3 {zeta} Subunit Contains a Phosphoinositide-Binding Motif That Is Required for the Stable Accumulation of TCR-CD3 Complex at the Immunological Synapse. J Immunol.

[40] Deford-Watts LM (2009). The cytoplasmic tail of the T cell receptor CD3 epsilon subunit contains a phospholipid-binding motif that regulates T cell functions. J Immunol.

[41] Minguet S, Swamy M, Alarcon B, Luescher IF, Schamel WW (2007). Full activation of the T cell receptor requires both clustering and conformational changes at CD3. Immunity.

[42] Wu W, Shi X, Xu C (2016). Regulation of T cell signalling by membrane lipids. Nature Reviews Immunology.

[43] Kress A (2013). Mapping the local organization of cell membranes using excitation-polarization-resolved confocal fluorescence microscopy. Biophysical journal.

[44] Benninger, R. K. Fluorescence Linear Dichroism Imaging for Quantifying Membrane Order. *Methods in Membrane Lipids*, 161–179 (2015). 10.1007/978-1-4939-1752-5_14 PMC4560120 25331136

[45] Lasserre R (2008). Raft nanodomains contribute to Akt/PKB plasma membrane recruitment and activation. Nat Chem Biol.

[46] Blouin CM (2016). Glycosylation-Dependent IFN-gammaR Partitioning in Lipid and Actin Nanodomains Is Critical for JAK Activation. Cell.

[47] Schacht J (1978). Purification of polyphosphoinositides by chromatography on immobilized neomycin. Journal of Lipid Research.

[48] Aharonovitz O (2000). Intracellular pH regulation by Na+/H+ exchange requires phosphatidylinositol 4, 5-bisphosphate. J Cell Biol.

[49] Temmerman K (2008). A direct role for phosphatidylinositol‐4, 5‐bisphosphate in unconventional secretion of fibroblast growth factor 2. Traffic.

[50] Ma, Y. *et al*. An intermolecular FRET sensor detects the dynamics of T cell receptor clustering. *Nature Communications* **8** (2017). 10.1038/ncomms15100 PMC5414349 28452360

[51] Ma Y (2017). A FRET sensor enables quantitative measurements of membrane charges in live cells. Nature Biotechnology.

[52] Xu Z, Stokoe D, Kane LP, Weiss A (2002). The inducible expression of the tumor suppressor gene PTEN promotes apoptosis and decreases cell size by inhibiting the PI3K/Akt pathway in Jurkat T cells. Cell Growth and Differentiation.

[53] Freeburn RW (2002). Evidence that SHIP-1 contributes to phosphatidylinositol 3, 4, 5-trisphosphate metabolism in T lymphocytes and can regulate novel phosphoinositide 3-kinase effectors. The Journal of Immunology.

[54] Liu B, Chen W, Evavold BD, Zhu C (2014). Accumulation of dynamic catch bonds between TCR and agonist peptide-MHC triggers T cell signaling. Cell.

[55] Liu B (2015). The cellular environment regulates *in situ* kinetics of T‐cell receptor interaction with peptide major histocompatibility complex. European journal of immunology.

[56] Sheng R (2016). Lipids regulate Lck protein activity through their interactions with the Lck Src homology 2 domain. Journal of Biological Chemistry.

[57] Sun Y, Dandekar RD, Mao YS, Yin HL, Wülfing C (2011). Phosphatidylinositol (4, 5) bisphosphate controls T cell activation by regulating T cell rigidity and organization. PLoS One.

[58] Thauland TJ, Hu KH, Bruce MA, Butte MJ (2017). Cytoskeletal adaptivity regulates T cell receptor signaling. Sci. Signal..

[59] Hui E, Vale RD (2014). *In vitro* membrane reconstitution of the T-cell receptor proximal signaling network. Nature structural & molecular biology.

[60] Drucker P, Pejic M, Grill D, Galla HJ, Gerke V (2014). Cooperative binding of annexin A2 to cholesterol- and phosphatidylinositol-4,5-bisphosphate-containing bilayers. Biophys J.

[61] Swamy M (2016). A Cholesterol-Based Allostery Model of T Cell Receptor Phosphorylation. Immunity.

[62] Dobbins J (2016). Binding of the cytoplasmic domain of CD28 to the plasma membrane inhibits Lck recruitment and signaling. Sci. Signal..

[63] Dieffenbach, C.W. & Dveksler, G. PCR primer. *A laboratory manual. Cold Spring Harbor, N*Y (1995).

[64] Carson RT, Vignali KM, Woodland DL, Vignali DA (1997). T cell receptor recognition of MHC class II-bound peptide flanking residues enhances immunogenicity and results in altered TCR V region usage. Immunity.

[65] Drevot P (2002). TCR signal initiation machinery is pre-assembled and activated in a subset of membrane rafts. Embo J.

[66] Xu K, Zhong G, Zhuang X (2013). Actin, spectrin, and associated proteins form a periodic cytoskeletal structure in axons. Science.

[67] Payrastre B (2004). Phosphoinositides: lipid kinases and phosphatases. Methods Mol Biol.

[68] Hamon Y (2000). ABC1 promotes engulfment of apoptotic cells and transbilayer redistribution of phosphatidylserine. Nat Cell Biol.

[69] Billaudeau C (2013). Probing the plasma membrane organization in living cells by spot variation fluorescence correlation spectroscopy. Methods Enzymol.

[70] Lenne PF (2006). Dynamic molecular confinement in the plasma membrane by microdomains and the cytoskeleton meshwork. Embo J.

[71] Mailfert S, Hamon Y, Bertaux N, He H-T, Marguet D (2017). A user’s guide for characterizing plasma membrane subdomains in living cells by spot variation fluorescence correlation spectroscopy. Methods in Cell Biology.

[72] Serge A, Bertaux N, Rigneault H, Marguet D (2008). Dynamic multiple-target tracing to probe spatiotemporal cartography of cell membranes. Nat Methods.

[73] Tsuji A, Ohnishi S (1986). Restriction of the lateral motion of band 3 in the erythrocyte membrane by the cytoskeletal network: dependence on spectrin association state. Biochemistry.

