# Author Correction: Phosphoinositides regulate the TCR/CD3 complex membrane dynamics and activation

**DOI:** 10.1038/s41598-020-69144-2

**Published:** 2020-07-23

**Authors:** Nassima Chouaki Benmansour, Kilian Ruminski, Anne-Marie Sartre, Marie-Claire Phelipot, Audrey Salles, Elise Bergot, Ambroise Wu, Gaëtan Chicanne, Mathieu Fallet, Sophie Brustlein, Cyrille Billaudeau, Anthony Formisano, Sébastien Mailfert, Bernard Payrastre, Didier Marguet, Sophie Brasselet, Yannick Hamon, Hai-Tao He

**Affiliations:** 1 Aix Marseille Univ, CNRS, INSERM, CIML, Centre d’Immunologie de Marseille-Luminy, Marseille, France; 2 Institut des Maladies Métaboliques et Cardiovasculaires, Inserm U1048, Université Toulouse 3, Toulouse, France; 3 Laboratoire D’Hématologie, Centre Hospitalier Universitaire de Toulouse, Toulouse, France; 4 Aix-Marseille Univ, CNRS, Centrale Marseille, Institut Fresnel, UMR 7249, 13397 Marseille, France; 5 Present Address: UTechS Photonic BioImaging (Imagopole) Citech, Institut Pasteur, 75724 Paris, France; 6 Present Address: Micalis Institute, INRA, AgroParisTech, Université Paris-Saclay, 78350 Jouy-en-Josas, France

Correction to: *Scientific Reports* 10.1038/s41598-018-23109-8, published online 21 March 2018

The original version of this Article contained a typographical error in the spelling of the author Nassima Chouaki Benmansour, which was incorrectly given as Nassima Chouaki-Benmansour. This has now been corrected in the PDF and HTML versions of the Article, and in the accompanying Supplementary Information file.

The Author Contributions section now reads:

“Y.H., N.C.B., K.R., A.-M.S., M.-C.P., A.S., E.B., A.W., G.C. designed the study, performed the experiments and analyzed the results. G.C., M.F., S.B., C.B., A.F., S.M. assisted with realization and interpretation of the experiments, and provided several reagents. B.P., D.M., S.B., Y.H. and H.-T.H. supervised and directed the research. Y.H., N.C.B. and H.-T.H. wrote the manuscript. All authors discussed the results and commented on the manuscript.”

