# Supplementary material for: Phosphoinositides regulate the TCR/CD3 complex membrane dynamics and activation
Source: Sci Rep. 2018 Mar 21;8:4966. doi: 10.1038/s41598-018-23109-8 (PMC5862878; doi:10.1038/s41598-018-23109-8)
Supplement: Supplementary file 1 — Supplementary Information [file 41598_2018_23109_MOESM1_ESM.pdf]

Supplementary Information

Phosphoinositides regulate the TCR/CD3 complex membrane dynamics and activation

Nassima Chouaki Benmansour[1], Kilian Ruminski[1], Anne-Marie Sartre[1], Marie-Claire Phelipot[1], Audrey Salles[1,5], Elise Bergot[1], Ambroise Wu[1], Gaëtan Chicanne[2], Mathieu Fallet[1] Sophie Brustlein[1], Cyrille Billaudeau[1,6], Anthony Formisano[1], Sébastien Mailfert[1], Bernard Payrastre[2,3], Didier Marguet[1], Sophie Brasselet[4], Yannick Hamon[1] & Hai-Tao He[1]

[1] Aix Marseille Univ, CNRS, INSERM, CIML, Centre d'Immunologie de Marseille-Luminy, Marseille, France

[2] Institut des Maladies Métaboliques et Cardiovasculaires, Inserm U1048, Université Toulouse 3, Toulouse, France

[3] Laboratoire d'Hématologie, Centre Hospitalier Universitaire de Toulouse, Toulouse, France

[4] Aix-Marseille Univ, CNRS, Centrale Marseille, Institut Fresnel, UMR 7249, 13397 Marseille, France

[5] Current address: UTechS Photonic BioImaging (Imagopole) Citech, Institut Pasteur, Paris, 75015, France

[6] Current address: Micalis Institute, INRA, AgroParisTech, Université Paris-Saclay, 78350 Jouy-en-Josas, France

Yannick Hamon & Hai-Tao He contributed equally to this work. Correspondence and requests for materials should be addressed to Y.H. (hamon@ciml.univ-mrs.fr) or H.-T.H. (he@ciml.univ-mrs.fr)

The Supplementary Information includes six supplementary figures (Figures S1 – S6) and one supplementary table (Table S1).

Figure S1

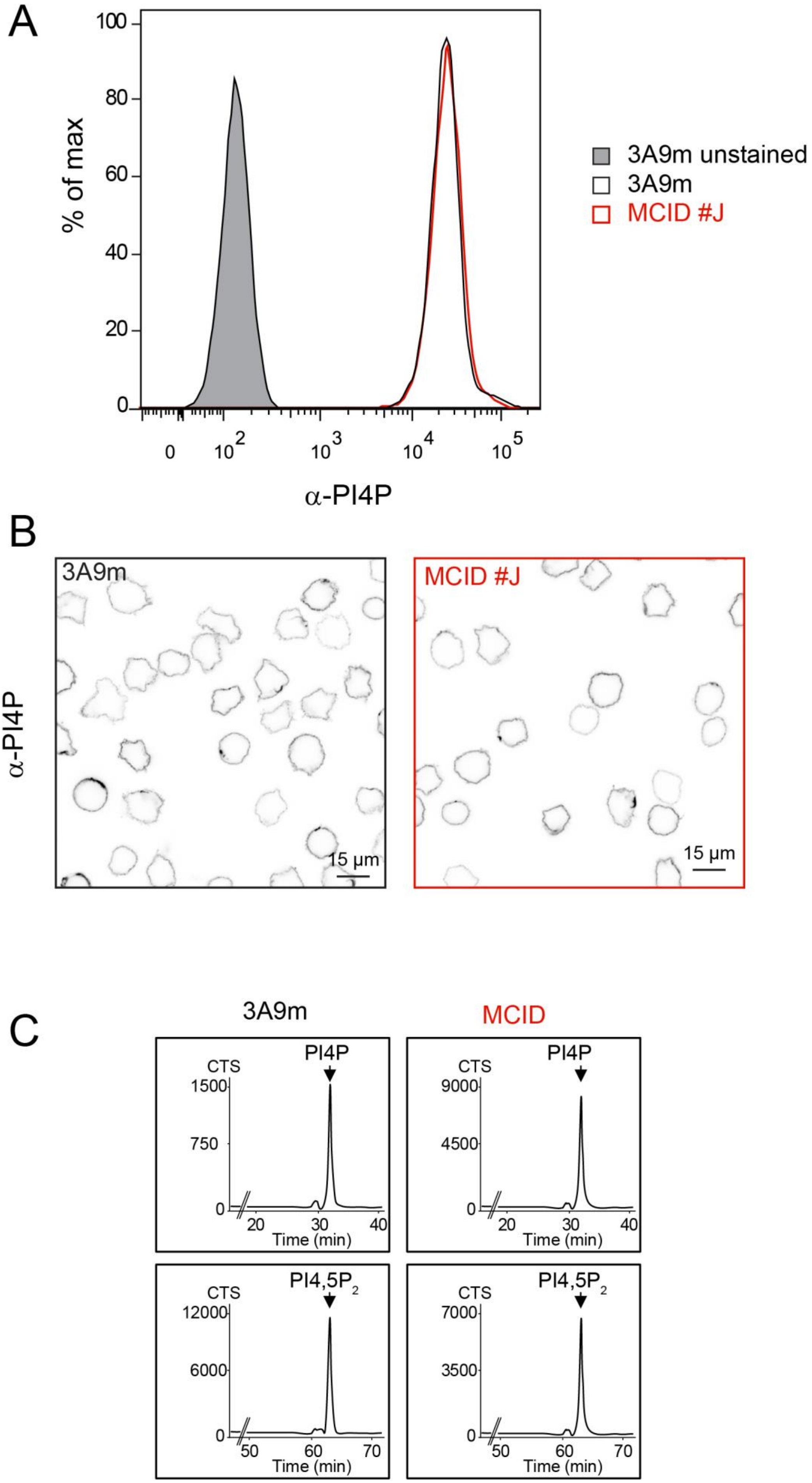
A
100
80
60
40
20
0
% of max
0
10^2
10^3
10^4
10^5
α-PI4P
3A9m unstained
3A9m
MCID #J
B
3A9m
MCID #J
α-PI4P
15 µm
15 µm
C
3A9m
MCID
PI4P
CTS
1500
750
0
20
30
40
Time (min)
PI4P
CTS
9000
4500
0
20
30
40
Time (min)
PI4,5P2
CTS
12000
6000
0
50
60
70
Time (min)
PI4,5P2
CTS
7000
3500
0
50
60
70
Time (min)

Figure S1: Analysis of PI4P levels upon MCID expression

(A) Flow cytometry analysis of the PI4P PM level in 3A9m and MCID #J cells, espectively. The unstained control is shown in gray.

(B) Confocal images of the anti-PI4P mAb labelled cells in (A).

(C) HPLC profile of PI4P and PI(4,5)P_2 extracted from [^{32}Pi]-orthophosphate labelled 3A9m WT and MCID cells, respectively (see Methods for details).

Figure S2

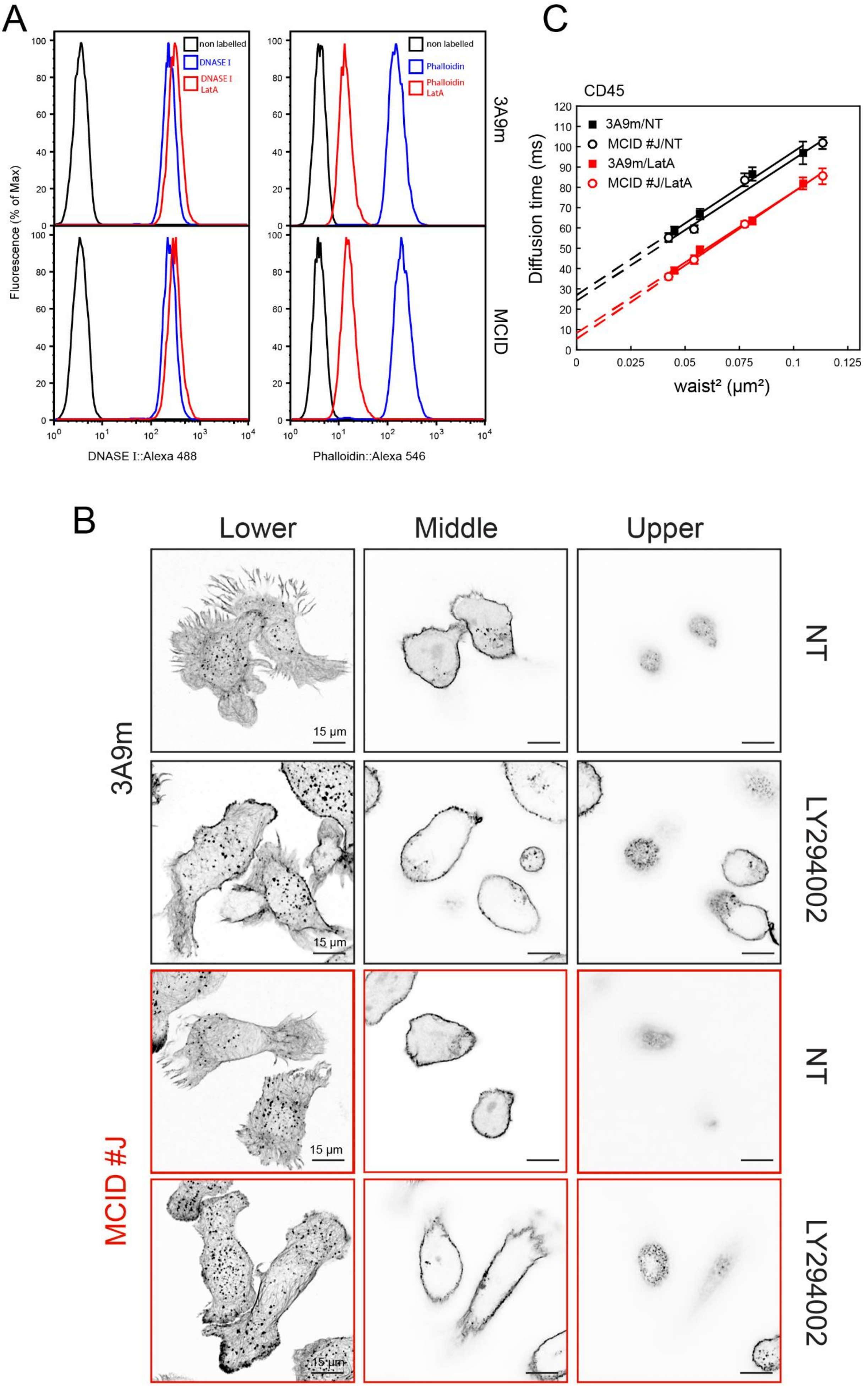

A
non labelled
DNASE I
DNASE I LatA
Phalloidin
Phalloidin LatA
3A9m
MCID
Fluorescence (% of Max)
DNASE I::Alexa 488
Phalloidin::Alexa 546
C
CD45
3A9m/NT
MCID #J/NT
3A9m/LatA
MCID #J/LatA
Diffusion time (ms)
waist² (µm²)
B
Lower
Middle
Upper
3A9m
MCID #J
NT
LY294002
15 µm

Figure S2: The MCID expression minimally affects the organization of the F-actin cortex

(A) Flow cytometry analysis with Alexa 488-conjugated DNASE 1 (left panels) and Alexa 546-conjugated phalloidin (right panels) of the untreated (in blue) or Latruncunlin A (that induces actin depolymerization, 2.5µM) treated (in red) 3A9m WT or MCID cells, respectively.

(B) Super-resolution microscopy of the actin cytoskeleton architecture of the antibody-immobilized 3A9m or MCID #J cells, treated or not with PI3K inhibitor LY294002 before being stained with Alexa 647-conjugated phalloidin and imaged by the confocal microscopy enhanced with airyscan technology. Lower, middle and upper optical sections are shown from a Z-stack acquisition (see Methods for details). These data, together with those shown in (A), depicted an overall preserved actin cortex architecture upon MCID expression. In addition, no difference in the actin cortex organization could be found between 3A9m and MCID #J cells after inhibition of the PI3K activity to deplete PI(3,4,5)P3.

(C) svFCS analysis of the PM lateral diffusion of CD45 labelled with Alexa 488::H193.16.3 Fab, in non-treated (in black) and Latruncunlin A-treated (in red) 3A9m or MCID #J cells, respectively. The lateral diffusion of CD45 in the PM of T cells is known to be constrained by the cortical actin cytoskeleton[1]. The highly sensitive svFCS technique[2,3] in combination with Latruncunlin A treament allowed establishing that the F-actin-constrained lateral diffusion for CD45 was unchanged upon MCID expression, indicating a normal cortical actin cytoskeleton in the MCID-expressing cells (see Methods for the details of svFCS analysis).

Figure S3

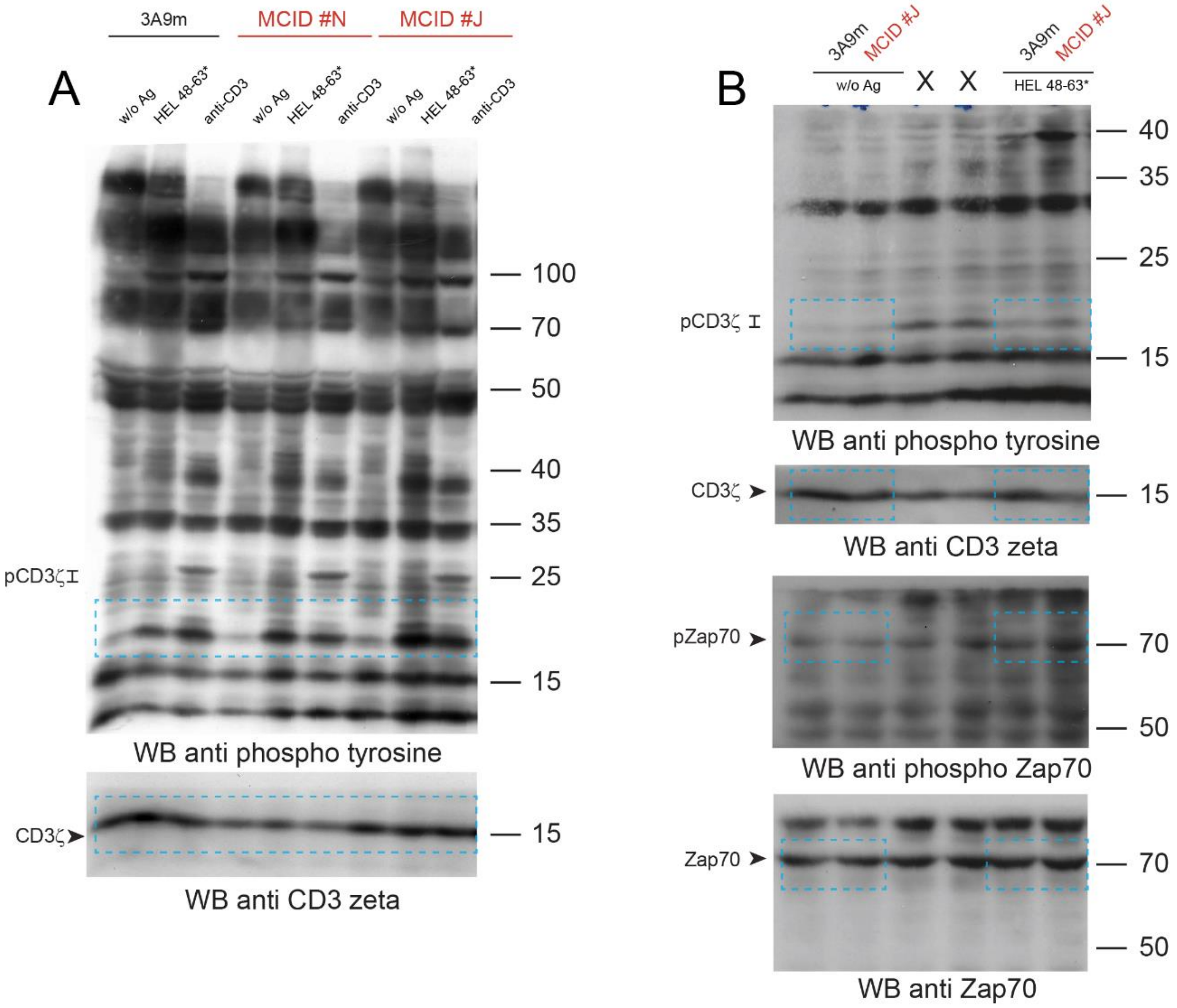

Figure S3: Uncropped images of immunoblots shown in Figure 2

(A) 3A9m mock-transfected WT cells or MCID #N or #J clones were seeded onto COS-A^k or COS-$A^k_{48\text{-}63}$* cells, or incubated with soluble anti-CD3ε mAb (145-2C11, 10 µg/ml), for 5 min at 37°C. Cell lysates were immunoblotted with an anti-p-Tyr mAb (4G10) to detect the phosphorylated isoforms of CD3ζ (p21 and p23). The bottom portion of the blot was stripped and reprobed with an anti-CD3ζ antibody to detect the (p16) CD3ζ molecule.

(B) The experiment was carried out same as that in (A), except that only the lower portion of the blot was analyzed with anti-p-Tyr mAb to detect the phosphorylated isoforms of CD3ζ (p21 and p23), while the upper portion of the blot was analyzed with an anti-p-Zap-70 antibody to detecet phosphorylation of Zap-70 (pTyr 319). The bottom portion of the blot was stripped and

reprobed with an anti-CD3ζ antibody to detect the (p16) CD3ζ molecule. The upper portion of the blot was stripped and reprobed with an anti-Zap-70 mAb to detect the Zap-70 molecule.

Figure S4

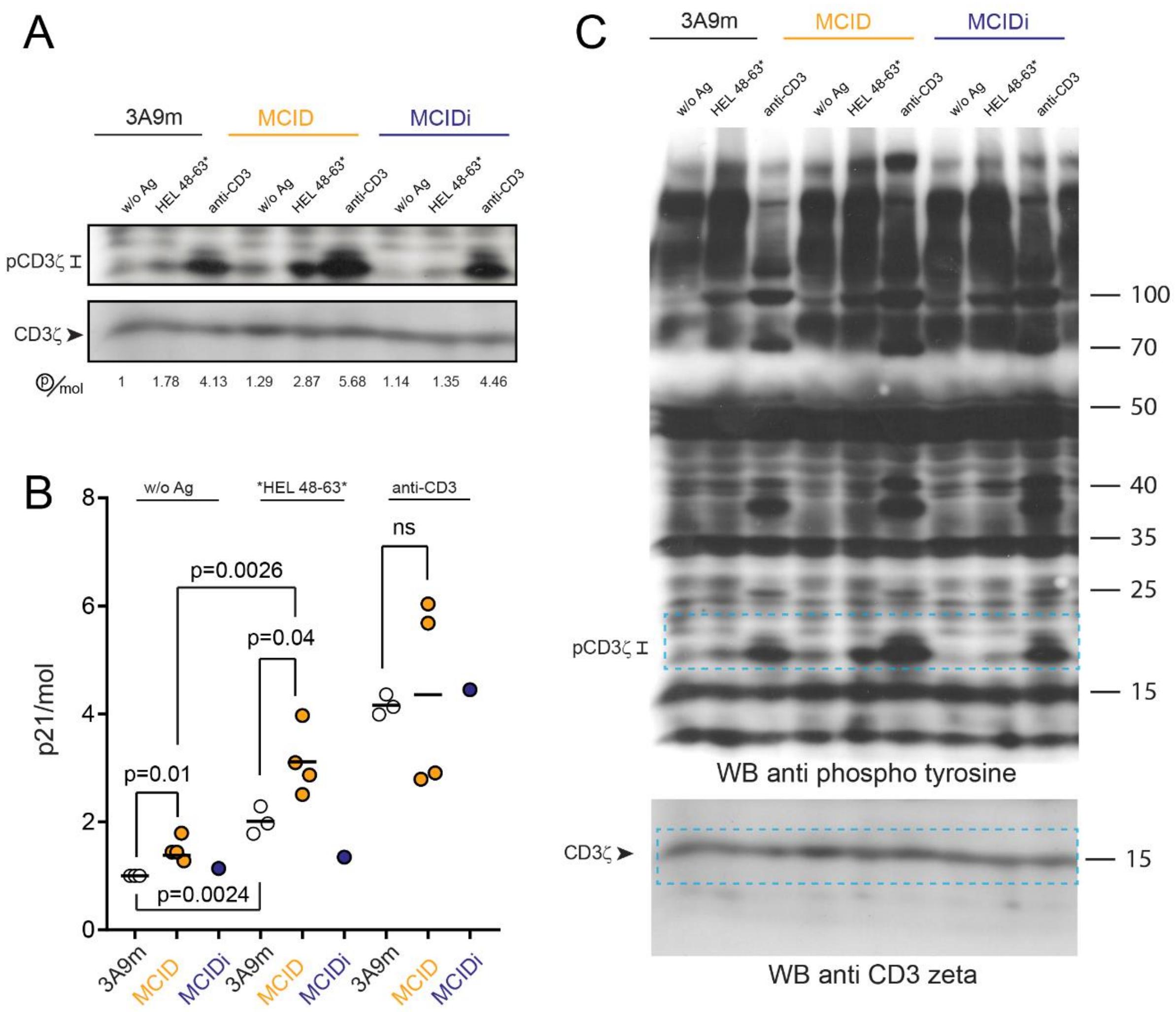

Figure S4: The increased TCR activation upon MCID expression

(A) 3A9m WT, MCID or MCIDi cells were seeded onto COS-A^k or COS-$A^k_{48\text{-}63}$* cells, or incubated with anti-CD3ε mAb (145-2C11, 10 µg/ml) for 5 min at 37°C. Cell lysates were immunoblotted with an anti-p-Tyr mAb (4G10) to detect the phosphorylated isoforms of CD3ζ (p21 and p23). The bottom portion of the blot was stripped and reprobed with an anti-CD3ζ antibody to detect the (p16) CD3ζ molecule.

(B) Statistical analysis of the p21/CD3ζ ratio from 3 independent immunoblots performed as in (A) for 3A9m, MCID and MCIDi quantification in (A) is plotted as a control.

(C) Uncropped version of the immunoblots shown in (A).

Figure S5

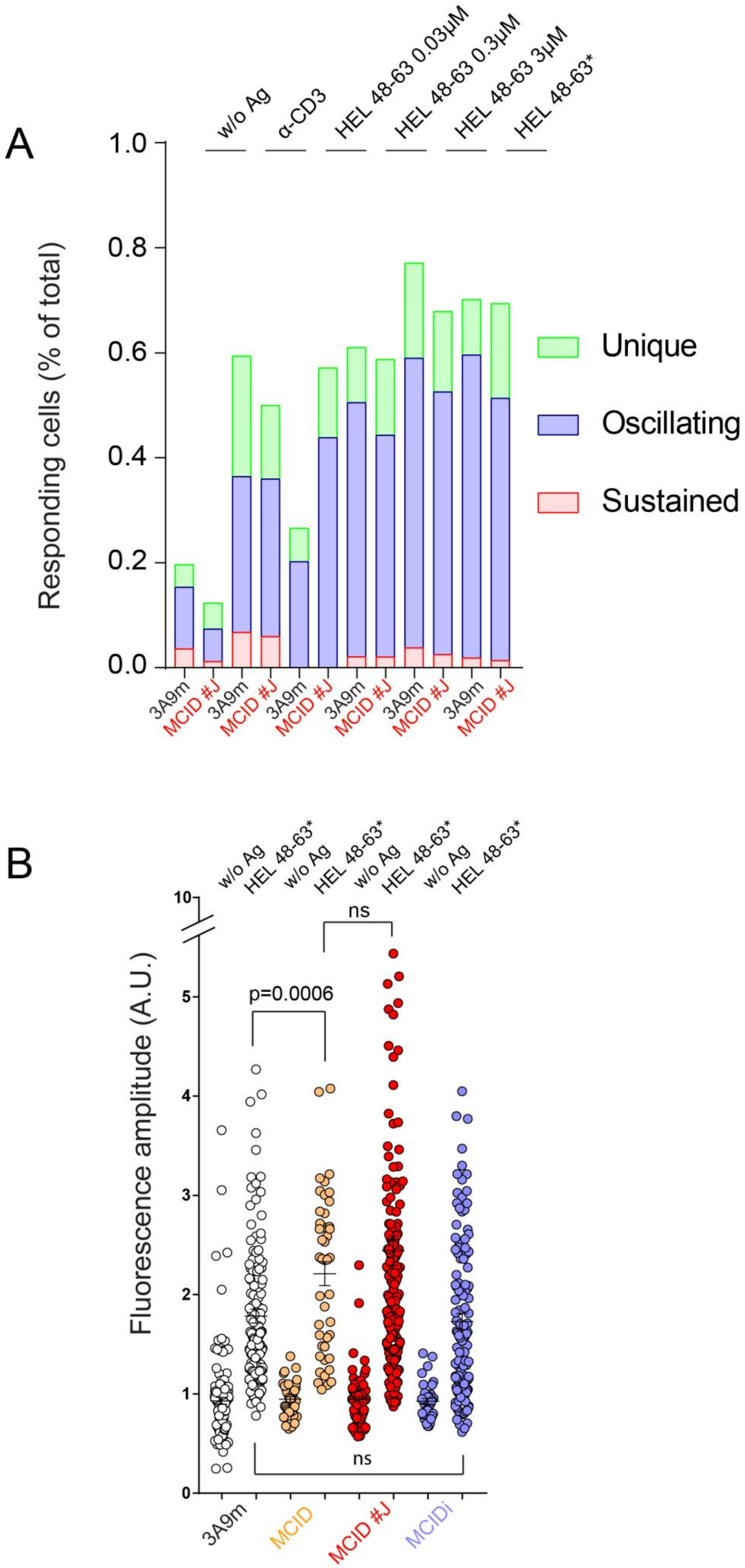
A
B
w/o Ag
α-CD3
HEL 48-63 0.03μM
HEL 48-63 0.3μM
HEL 48-63 3μM
HEL 48-63*
Responding cells (% of total)
1.0
0.8
0.6
0.4
0.2
0.0
Unique
Oscillating
Sustained
3A9m
MCID #J
HEL 48-63*
Fluorescence amplitude (A.U.)
10
5
4
3
2
1
0
ns
p=0.0006
MCID
MCIDi

Figure S5: The increased TCR-mediated Ca^{2+} store release upon MCD expression

(A) Percentage of cells over the activation threshold in the calcium imaging experiment of Fig. 2d.

(B) Comparative analysis of the fluorescence amplitude of individual cells over the threshold in 3A9m WT, 3A9m MCID, MCID #J and MCIDi cells upon incubation with COS-A^k cells or COS-$A^k_{48\text{-}63}$*.

Figure S6

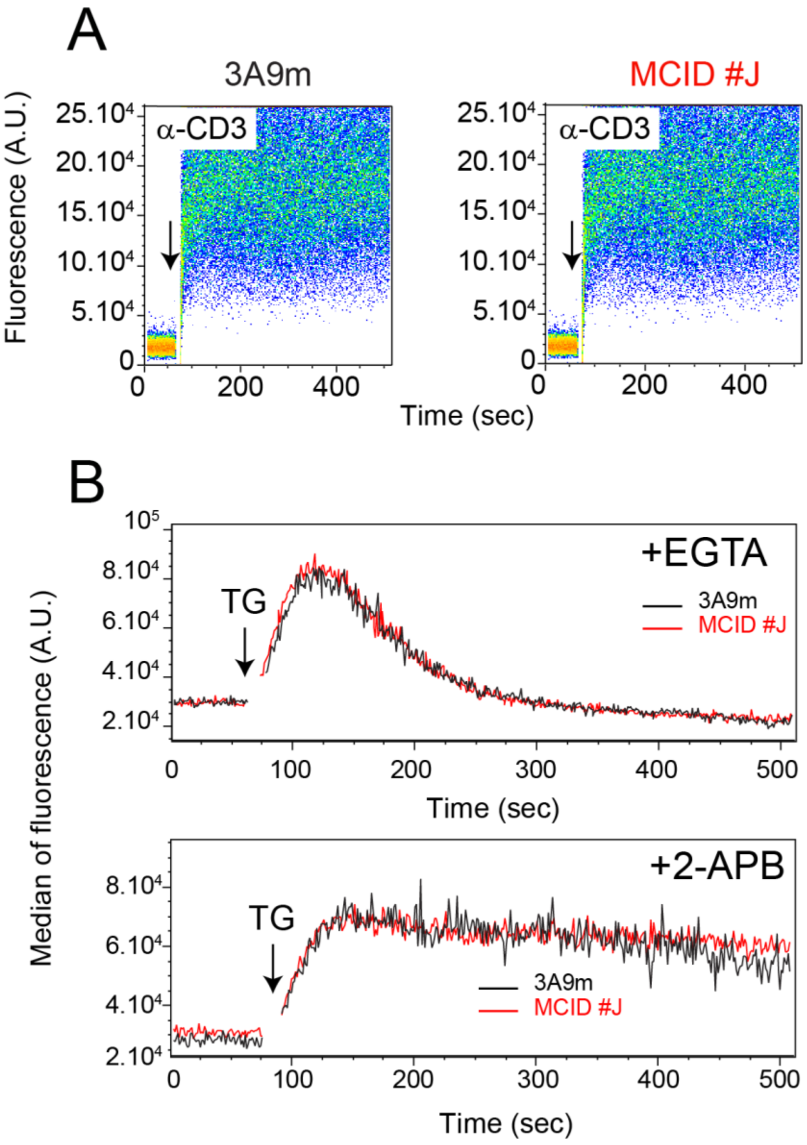

Figure S6: Flow cytometry analysis of soluble anti-CD3 mAb binding and the Ca^{2+} store release stimulted by thapsigargin

(A) Alexa 488-conjugated anti-CD3ε mAb binding kinetics on 3A9m (left panel) or MCID #J (right panel) cells were analyzed by flow cytometry and plotted over time as indicated in Methods. Autofluorescence was recorded for 1 min (and considered as the baseline) prior to addition of soluble fluorescent anti-CD3ε mAb (5 µg/ml).

(B) Calcium mobilization response upon stimulation with thapsigargin. Cells were stimulated upon addition of 0.1 µM thapsigargin (arrow) in the presence of 1 mM EGTA (upper panel) or 10 µM 2-APB (lower panel). Median of fluorescence are expressed as a function of time.

#	Construct		Oligos sequences	Strategy	Cloned into	Sites	Amplification matrix
2	Lck1-12::mCherry::INP54d (MCID)	For	GTC GTA ACA ACT CCG C	PCR overlap extension	pEGFPm-N2	EcoRI/NotI	pEGFP-N2m Lck1-12 (Cf Lasserre, R. Nat Chem Biol, 20
3		Rev	CCC GCG CCG GCT CCT GCG GAT CCC CTT GTA CAG CTC GTC CAT				
4		For	GGG ATC CGC AGG AGC CGG CGC GGG ATG AAC AAA ACG AAT TGG A				pCDNA3 lyn::EGFP::INP54 (gift from Jaques Nunes)
5		Rev	AAA GGA AAA AA GCGGCCGC TTA CGG CAC TGG CGT CC				
6	Lck1-12::mCherry::INP54d D311G	For	C AAT GCT CTA TTA TTT AGC GGT CAT CAA CCC GTT AAT C	Quikchange mutagenesis	NA	NA	MCID
7		Rev	G ATT AAC GGG TTG ATG ACC GCT AAA TAA TAG AGC ATT G				
8	Lck1-12::mCherry::INP54d D311G D280A (MCI	For	CCG TCG TGG TGC GCT AGG ATT CTC TAC AAA AGC	Quikchange mutagenesis	NA	NA	Lck1-12::mCherry::INP54 D311G
9		Rev	GCT TTT GTA GAG AAT CCT AGC GCA CCA CGA CGG				
10	CD3e::EGFPm	For	AAG AGC TTC GAA TT C ACC ATG CGG TGG AAC ACT TTC	PCR *in* -fusion cloning	pEGFPm-N2	EcoRI/BamHI	mouse CD3 epsilon cDNA (gift from Bent Rubin)
11		Rev	AC CGG CCG GTG GAT CCC GAC TGC TCT CTG ATT CAG				
12	CD3e::EGFPm R183S	Rev	AC CGG CCG GTG GAT CCC GAC TGC ACT CTG ATT CAG	PCR *in* -fusion cloning	pEGFPm-N2	EcoRI/BamHI	idem
13	CD3e::EGFPm Δ N181*	Rev	AC CGG CCG GTG GAT CCC CAG GCC AGA ATA CAG GT	PCR *in* -fusion cloning	pEGFPm-N2	EcoRI/BamHI	idem
14	CD3e::EGFPm Δ K171*	Rev	AC CGG CCG GAC GTG GAT CCC GCG GAT GGG CTC ATA G	PCR *in* -fusion cloning	pEGFPm-N2	EcoRI/BamHI	idem
15	CD3e::EGFPm Δ N163*	Rev	AC CGG CCG GTG GAT CCC GGG AAC AGG TGG TGG C	PCR *in* -fusion cloning	pEGFPm-N2	EcoRI/BamHI	idem
16	CD3e::EGFPm Δ P158*	Rev	AC CGG CCG GTG GAT CCC CCG CTC CTT GTT TTG CC	PCR *in* -fusion cloning	pEGFPm-N2	EcoRI/BamHI	idem
17	CD3e FF::EGFPm	For	CCC AAC CCA GAC TTT GAC CCC ATC CGC AAA GGC CAG CGG GAC CTG TTT TCT GGC CTG	Quikchange mutagenesis	NA	NA	idem
18		Rev	CAG GCC AGA AAA CAG GTC CCG CTG GCC TTT GCG GAT GGG CTC AAA GTC TGG GTT GGG				
19	CD3eCD:: EGFPm	For	ACGC GTC GAC CGCCACC ATG GTG AGC AAG AAT AGG AAG GC	PCR cloning	pEGFPm-N2	SalI/BamHI	idem
20		Rev	CGCT G GAT CCC GAC TGC ACT CTG ATT CAG				
21	myr::EGFPm	For	AATTC CGCC ACC ATG GGC AGT GTC AGC AGC TCA AAC CCT GAA GAT GAC	Annealed oligos	pEGFPm-N2	EcoRI/SalI	NA
22		Rev	GAT TC ATC TTC AGG GTT TGA GCT GCT GAC ACT GCC CAT GGT GGCG G				
23	myr::CD3eCD::EGFPm (Lck1-12_Δ_Palm_Palm referred as myr)	For	CGG GAATTC CGCC ACC ATG GGC AGT GTC AGC AGC TCA AAC CCT GAA GAT GAC TGG AGC AAG AAT AGG AAG	PCR cloning	pEGFPm-N2	EcoRI/SalI	mouse CD3 epsilon cDNA (gift from Bent Rubin)
24		Rev	ACG G TCG ACT GCC CCC TCC ACC GAC TGC ACT CTG ATT CAG				
25	Lck 1-12::CD3eCD::EGFPm	For	TAC CAT ATG GGC TGT GTC TGC AGC TCA AAC CCT	Quikchange mutagenesis	NA	NA	myr::CD3eCD::mEGFP
26		Rev	AGG GTT TGA GCT GCA GAC ACA GCC CAT GGT A				
27	Lck1-1-12::CD3eCD_KS::EGFPm	For	GAA GAT GAC TGG AGC AGC AAT AGT AGC GCC TCG GCC AGC CCT GTG ACC TCA GGT ACC GGT GCT GGT AGC AGG CCC	Quikchange mutagenesis	NA	NA	Lck1-1-12::CD3eCD::EGFPm
28		Rev	GGG CCT GCT ACC AGC ACC GGT ACC TGA GGT CAC AGG GCT GGC CGA GGC GCT ACT ATT GCT GCT CCA GTC ATC TTC				
29	Lck::EGFP	For	TAA TAC GAC TCA CTA TAG G	PCR overlap extension	pDNA 3.1(+)	Hind III/NotI	pCDNA 3 Lck
30		Rev	GCT TCC ACC GCC TCC AGG CTG GGG CTG GTA				
31		For	GGA GGC GGT GGA AGC ATG GTG AGC AAG GGC G				pEGFP-N2m Lck1-12 (Cf Lasserre, R. Nat Chem Biol, 20
32		Rev	AAG AAT GCGGCCGC T TTA CTT GTA CAG CTC GTC				
33	Lactadherin GFP	For	GCA GAC AGATCT TGC ACT GAA CCC CTA G	PCR	pEGFPm-C1	BglII/EcoRI	Bovine Lactadherin cDNA
34		Rev	GCA GAC GAATTC CTA ACA GCC CAG CAG				
35	(PH PLCδ)*	For	C CGG AAT TC T ATG GAC TCG GGC CGG	PCR	pEGFPm-C2 PH_PLCδ	EcoRI/BamHI	PH PLCδ cDNA (Gift from Georges Bismuth)
36		Rev	CGC GGA TCC TCT GCC CCC TCC ACC CTG GAT GTT GAG CTC CT				
37	Lck1-12::EGFPm::Hras Cterm	For	GTAC AAG CCC GGG TGC ATG AGC TGC AAG TGT GTG CTC TCC TAA AGC	Annealed oligos	pEGFPm-N2 Lck1-12	BsrGI/NotI	NA
38		Rev	GGCCGCT TTA GGA GAG CAC ACA CTT GCA GCT CAT GCA CCC GGG CTT				
39	Lck1-12::EGFPm::MARCKS ED	For	G TAC AAG TTC GGA CTC GCT TCT AAG AAT AGG AAG GCC AAG GCC AAG CCT GTG ACC CGA GGA ACC GGT GCT GGT AGC AGG CCC AGA GGG CAA AAC AAG GAG CGG TAA GC	Annealed oligos	pEGFPm-N2 Lck1-12	BsrGI/NotI	NA
40		Rev	GG CC GC TTA CCG CTC CTT GTT TTG CCC TCT GGG CCT GCT ACC AGC ACC GGT TCC TCG GGT CAC AGG CTT GGC CTT GGC CTT CCT ATT CTT AGA AGC GAG TCC GAA CTT				
41	Lck1-12::EGFPm::CD3eBRS	For	G TAC AAG CCG AAG AAT AGG AAG GCC AAG GCC AAG CCT GTG ACC CGA GGA ACC GGT GCT GGT AGC AGG CCC AGA GGG CAA AAC AAG GAG CGG TAA GC	Annealed oligos	pEGFPm-N2 Lck1-12	BsrGI/NotI	NA
42		Rev	GG CC GC TTA CCGCTCCTTGTTTTGCCCTCTGGGCCTGCTACCAGCACCGGTTCCTCGGGTCACAGGCTTGGCCTTGGCCTTCCTATTCTT CGG CTT				
43	Lck1-12::EGFPm::PHPLCδ	For	ATG GAC GAG CTG TAC AAG GAC TCG GGC CGG GAC	PCR	pEGFPm-N2 Lck1-12	BsrGI/NotI	PH PLCδ cDNA (Gift from Georges Bismuth)
44		Rev	TCT AGA GTC GCG GCC GCT TTA CTG GAT GTT GAG CTC C				
45	Lck1-12::EGFPm::Lactadherin C2	For	ATG GAC GAG CTG TAC AAG TCC GGA CTC AGA TCT TG	PCR	pEGFPm-N2 Lck1-12	BsrGI/NotI	Bovine Lactadherin cDNA
46		Rev	TCT AGA GTC GCG GCC GCT CTA ACA GCC CAG CAG C				
47	Lck1-12::EGFPm::Kras Cterm	For	G TAC AAG TTC GGA CTC GCT TCT AAG ATG AGC AAA GAT GGT AAA AAG AAG AAA AAG AAG TCA AAG ACA AAG TGT GTA ATT ATG TAA GC	Annealed oligos	pEGFPm-N2 Lck1-12	BsrGI/NotI	NA
48		Rev	GG CC GC TTA CAT AAT TAC ACA CTT TGT CTT TGA CTT CTT TTT CTT CTT TTT ACC ATC TTT GCT CAT CTT AGA AGC GAG TCC GAA CTT				

***Table S1*: Summary of molecular biology constructs used in this study.**

The sequence, the cloning strategy, the type of vector and the amplification matrix are summarized for each oligo corresponding to different constructs cited in this article.

Supplementary references

1. Cairo, C.W. et al. Dynamic regulation of CD45 lateral mobility by the spectrin-ankyrin cytoskeleton of T cells. *J Biol Chem* **285**, 11392-401 (2010).
2. He, H.T. & Marguet, D. Detecting nanodomains in living cell membrane by fluorescence correlation spectroscopy. *Annu Rev Phys Chem* **62**, 417-36 (2011).
3. Blouin, C.M. et al. Glycosylation-Dependent IFN-gammaR Partitioning in Lipid and Actin Nanodomains Is Critical for JAK Activation. *Cell* **166**, 920-34 (2016).
